# Neoadjuvant immunotherapy for DNA mismatch repair proficient/microsatellite stable non-metastatic rectal cancer: a systematic review and meta-analysis

**DOI:** 10.3389/fimmu.2025.1523455

**Published:** 2025-01-27

**Authors:** Huan Zhang, Jing Huang, Huanji Xu, Nanhao Yin, Liyan Zhou, Jianxin Xue, Min Ren

**Affiliations:** ^1^ Division of Thoracic Tumor Multimodality Treatment, Cancer Center, West China Hospital, Sichuan University, Chengdu, Sichuan, China; ^2^ Department of Ultrasound, West China Hospital, Sichuan University, Chengdu, Sichuan, China; ^3^ Abdominal Oncology Ward, Division of Radiation Oncology, Cancer Center, West China Hospital, Sichuan University, Chengdu, Sichuan, China; ^4^ Laboratory of Clinical Cell Therapy, West China Hospital, Sichuan University, Chengdu, Sichuan, China; ^5^ Department of Radiation Oncology, Cancer Center, West China Hospital, Sichuan University, Chengdu, Sichuan, China

**Keywords:** neoadjuvant immunotherapy, non-metastatic rectal cancer, mismatch repair-proficient/microsatellite stable, meta-analysis, efficacy

## Abstract

**Background:**

Neoadjuvant immunotherapy (NIT) has been endorsed by clinical guidelines for the management of DNA mismatch repair deficiency/microsatellite instability-high (dMMR/MSI-H) locally advanced rectal cancer (LARC). Nonetheless, the therapeutic efficacy of NIT in mismatch repair-proficient/microsatellite stable (pMMR/MSS) non-metastatic rectal cancer (RC) remain pending matters. Therefore, a meta-analysis was carried out to assess the efficacy and safety of NIT in patients with non-metastatic pMMR/MSS RC.

**Methods:**

PubMed, Embase, Web of Science, the Cochrane Library, ClinicalTrials.gov, ASCO and ESMO were searched to obtain related studies up to July 2024. Two reviewers independently screened the included articles and extracted the pertinent data. The risk of publication bias was assessed by Begg or Egger tests and in cases of publication bias, the trim and fill method was applied. Heterogeneity was assessed using *I ^2^* statistics.

**Results:**

Thirteen articles including 582 eligible patients were analyzed. The pooled pCR, MPR, cCR and anus preservation rate were 37%, 57%, 26% and 77% separately and the incidence of irAEs≥3 grades and TRAEs≥3 grades were 3% and 29%, respectively. Non-metastatic pMMR/MSS RC receiving the short-course radiotherapy (SCRT) in neoadjuvant setting exhibited superior pooled pCR and MPR than long-course radiotherapy (LCRT) without upregulating the incidence of adverse effects. Furthermore, patients with MSS RC underwent neoadjuvant treatment with anti-PD-1 inhibitors demonstrated higher pooled pCR, MPR, cCR compared to those receiving PD-L1 inhibitors. Additionally, yielded improved pooled MPR and anal preservation rates compared to sequential immuno-radiotherapy (63.4% vs 51.2% and 88.5% vs 69.9%), without raising the incidence of irAEs≥3 grade. Interestingly, RC patients with lymph node metastasis showed a higher pooled pCR than those without lymph node metastasis (43% vs 35%).

**Conclusion:**

NIT was linked to favorable response rates and anal preservation, alongside an acceptable safety profile. Non-metastatic pMMR/MSS RC patients receiving SCRT, PD-1 inhibitors, or concurrent immuno-radiotherapy in the neoadjuvant setting exhibited enhanced outcomes. This meta-analysis provides evidence for further exploration and application of NIT in non-metastatic pMMR/MSS RC and highlights the potential for organ preservation with this approach. The relatively small sample size and the uneven quality of included studies may have had some impact on the generality of the results. Therefore, further analysis with a higher number of high-quality studies is needed to verify the conclusions.

**Systematic review registration:**

https://inplasy.com/, identifier: INPLASY202470110.

## 1 Introduction

Ranking second in cause of mortality and third in incidence of malignancy globally, colorectal cancer (CRC) brings a serious threat to human health with a persistent upward trend in incidence and fatalities, among which, rectal cancer (RC) accounts for approximately 33.3% of all the diagnosed cases (1). Although notable medical advancements had been achieved in the past few years, the locally advanced rectal cancer (LARC) was still a tricky disease to management with increased incidence, high propensity of local recurrence and distant metastasis (2) and prevalence in younger populations (3).

Total neoadjuvant therapy (TNT) refers to the perioperative treatment for LARC where the majority or entirety of postoperative adjuvant chemotherapy is administered prior to surgical intervention, in conjunction with concurrent chemoradiotherapy (CRT). In the Spanish phase II randomized GCR-3 trial, pathologic complete response (pCR) in the TNT group and neoadjuvant chemoradiotherapy (nCRT) group was not significantly different (13.5% vs 14.3%), yet the TNT cohort exhibited superior treatment adherence (91% vs 54%) (4). A retrospective analysis from Memorial Sloan Kettering Cancer Center (MSK) revealed that patients with LARC receiving TNT experienced higher pCR rates than those undergoing conventional chemoradiotherapy (5). Consequently, TNT is recommended as one of the standard treatments for LARC. Besides, for LARC patients achieving a complete clinical response (cCR) after neoadjuvant treatment, the conservative Watch and Wait (WW) strategy may offer comparable survival outcomes to surgical intervention (6). Nonetheless, despite the progress made with TNT for LARC, various limitations persist, including a distant metastasis rate exceeding 20% within three years, a postoperative pCR rate less than 30%, heightened toxicity from radiotherapy and chemotherapy, and poor long-term survival prospects, which somewhat restrict clinical application.

In the last decade, cancer immunotherapy—encompassing antibody therapy, cellular immunotherapy, and cytokine therapy—has transformed the oncology treatment landscape, yielding promising clinical results across a wide array of malignancies (7). However, only a minority of patients with specific molecular profiles derive substantial benefit from immunotherapy (8). Chromosome translocations and genomic instability are hallmarks in cancer development. The DNA mismatch repair (MMR) system is crucial for preserving DNA integrity, with DNA mismatch repair deficient/microsatellite instability-high (dMMR/MSI-H) defined by mutation status in microsatellite alleles. Characterized by high tumor mutational burden (TMB), abundant tumor infiltrating lymphocytes (TILs) and non-synonymous mutations, MSI-H/dMMR tumors often show greater response rate for immune checkpoint inhibitors (ICIs) treatment. Conversely, the vast majority of patients with mismatch repair proficient/microsatellite stable (pMMR/MSS), which feature low TMB and limited T cell infiltration, tend to show decreased sensitivity or resistance to ICIs (8, 9).

In addition to damaging cancer cells directly, irradiation also exerts immunostimulant properties by enhancing the cytotoxic activity of NK cells and fostering the accumulation of CD8+ cytotoxic T lymphocytes and tumor-associated M1 macrophages within the tumor microenvironment. The concomitantly used immunotherapy can potentiate the activity of immune cells, resulting in significant neoplastic cell destruction or exhibiting synergistic antitumor effects in combination with radiotherapy (10, 11), as evidenced in studies involving triple-negative breast cancer, small cell lung cancer, and other tumors (12).

Building on these principles, numerous studies have investigated the clinical advantages of neoadjuvant immunotherapy (NIT) in CRC. The KEYNOTE-016 trial found that metastatic CRC and other solid tumors exhibiting the dMMR/MSI-H phenotype significantly benefit from programmed death ligand-1 (PD-L1) monoclonal antibody immunotherapy (13). Therefore, guidelines recommended immunotherapy for the treatment of dMMR/MSI-H metastatic CRC. Although only a very small part (less than 10%) of RC can be classified as dMMR/MSI-H category, NIT could result in a higher complete response (CR) rate than nCRT with fewer adverse effects on sphincter, reproductive organs and sexual function in those group of population (14), and the latest ASCO guideline recommended the NIT and the first-line treatment option for dMMR/MSI-H LARC (15).

Despite advancements in the treatment of dMMR/MSI-H tumors, consensus on the clinical efficacy of NIT for pMMR/MSS non-metastatic RC remains elusive. Besides, there are only few published RCT studies reporting the clinical efficacy and safety of NIT in MSS RC, most of the on-going trials are single-arm prospective trials and the variations about intervention methods in these published and ongoing trials also impede the clinical application of NIT in MSS RC. Given the rising demand to achieve tumor regression, anus preservation and more satisfactory long-term survival outcomes through “increasing efficiency and decreasing toxicity” therapeutic strategy in RC patients recent years, the traditional nCRT or TNT treatment model reached an impasse. Therefore, we conducted this systematic review and meta-analysis to assess the effect and safety of immunotherapy-based neoadjuvant treatment in non-metastatic pMMR/MSS RC, aiming to offer novel management options for these patient populations and provide support for future study.

## 2 Methods

This systematic review with meta-analysis was executed in compliance with the Preferred Reporting Items for Systematic Reviews and Meta-Analyses (PRISMA) guidelines (16). The selection criteria were established based on the PICOS (population, intervention, comparison, outcome, and study design) framework.

### 2.1 Search strategy

We conducted comprehensive searches of several online databases for eligible trails from inception to July 2024, including PubMed, Embase, Web of Science and the Cochrane Library. Additionally, ClinicalTrials.gov, ASCO and ESMO also were searched for potential unpublished findings. Keywords used for the search included “rectal cancer” “neoadjuvant immunotherapy” “PD-1 inhibitors” “PD-L1 inhibitors” and “neoadjuvant therapy”. To ensure comprehensive coverage, references from original studies and literature reviews were also examined. Details of the search methodology could be available in **Supplementary File 1**.

### 2.2 Inclusion and exclusion criteria

Inclusion criteria were as follows: 1) adults with primary cancer of pMMR/MSS RC; 2) non-metastatic disease; 3) immunotherapy (programmed cell death protein 1 (PD-1) inhibitor, programmed cell death 1 ligand 1 (PD-L1) inhibitor or cytotoxic T-lymphocyte–associated protein 4 (CTLA-4) inhibitor) used during neoadjuvant therapy period; 4) reporting 10 or more cases and 5) single-arm study, cohort or prospective study, retrospective study and RCTs.

Exclusion criteria included: 1) letters to the editor, and editorials, reviews, animal studies, case reports and study protocol; 2) articles lacking related data; 3) involving patients diagnosed with colorectal cancer (CRC) or other malignancies without distinct findings, 4) metastatic CRC or RC, 5) absence of immunotherapy in the neoadjuvant setting.

### 2.3 Efficacy indicators

The outcomes evaluated in these studies were the pathological complete response (pCR), major pathological response (MPR), clinical complete response (cCR) and the anal preservation rates.

### 2.4 Quality assessment

Most trials included in our analysis were single-arm studies, therefore, we evaluated the quality of the research using the methodological index for non-randomized studies (MINORS), which, was used for the quality assessment of non-randomize studies (17). Study qualities were classified as poor (0–12), or good (13–16) based on MINORS scores, and any discrepancies were resolved through consensus.

### 2.5 Data extraction

Two investigators (Huan Zhang and Jing Huang) independently extracted relevant data from the included studies, including characteristic data from the study (first author, publication year, country/region of the patient, study type, sample size, gender, patient age, Eastern Cooperative Oncology Group (ECOG) performance status, distance from primary tumor to anal verge, clinical stage, clinical T category, clinical N category, type of radiotherapy, intervention methods, type of checkpoint inhibitor) and statistical data (pCR, MPR, cCR, anus preservation rate, incidence of TRAEs and irAE≥3 grades). The details of TRAEs, irAEs and clinical stage were shown in the **Supplementary Tables 1**–**3**, respectively. Where necessary, corresponding authors were contacted for additional information.

### 2.6 Statistical analysis

The meta-analysis was performed using Stata/MP 14.0 software (StataCorp LP, College Station, TX). A p value<0.05 was considered statistically significant. Heterogeneity between studies was categorized as low (*I^2^* <50%) or high (*I^2^* >50%) using the Cochran Q chi-square test and *I^2^* statistics. Random effects models and fixed effects model were used to analyze the data with huge heterogeneity (*I* ^2^≥50%) and little heterogeneity (*I* ^2^<50%), respectively. Subgroup analyses were conducted based on clinical factors to reduce heterogeneity. The identification of potential bias was accomplished by evaluating the asymmetry of the plot and Egger or Begg tests.

## 3 Results

### 3.1 Study selection and characteristics

**Figure 1:**
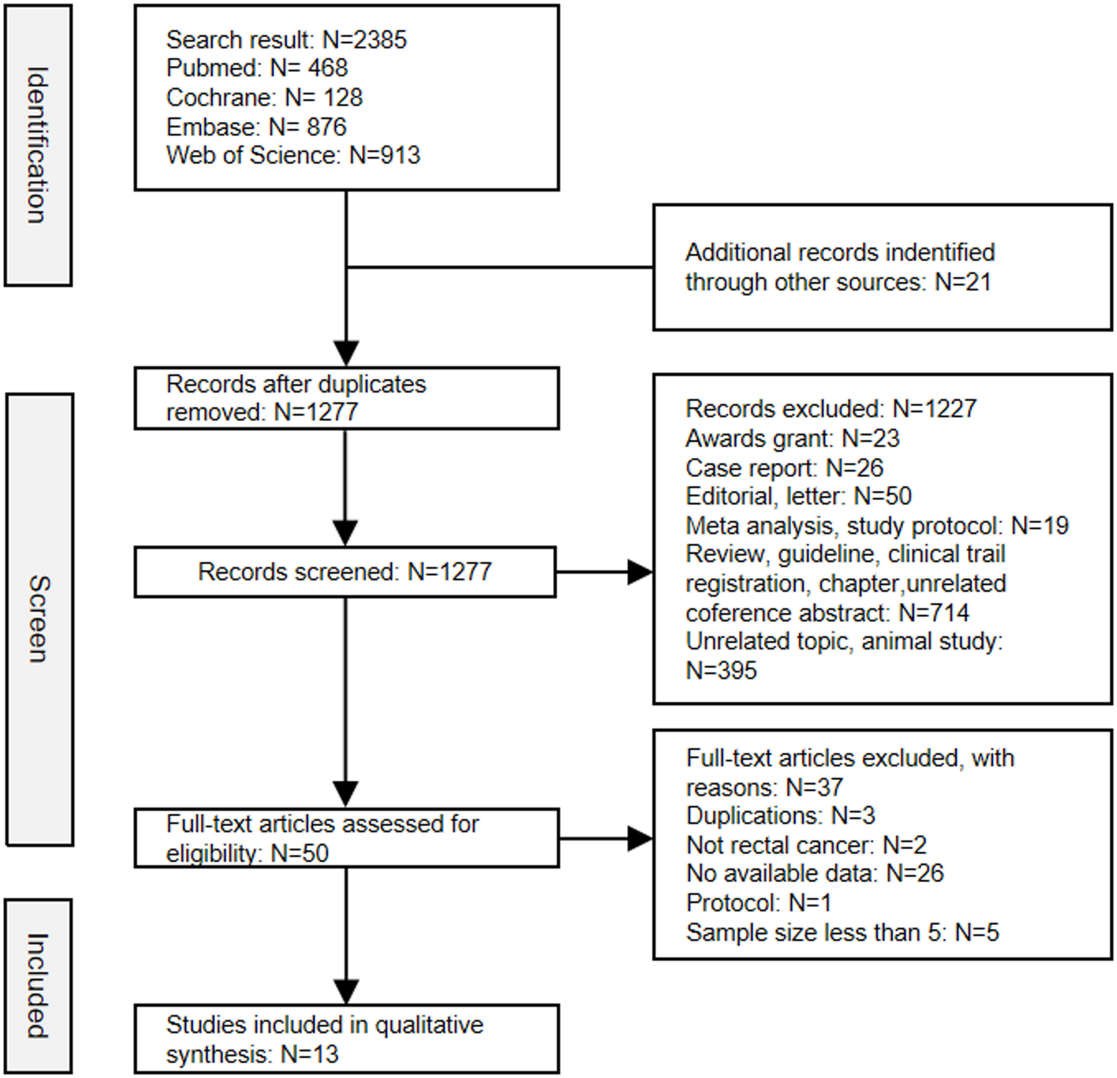
Flow diagram of the literature search in this meta-analysis.

After screening the title, abstract and full-text, a total of thirteen studies comprising 582 patients were ultimately included in this analysis (18–30). The selection process was conducted in accordance with the PRISMA flowchart guidelines (**Figure 1**). Of the studies included, seven were published as full papers and six were presented as conference abstracts. Among these studies included, four were randomized controlled trials (RCTs) and the remaining nine were prospective single-arm studies. The MINORS score system evaluated all studies as having good quality (**Supplementary Table 4**). The principal characteristics of studies included in this meta-analysis were summarized in **Table 1**.

**Table 1:** Characteristic of included studies.

First author, year	Region	Sample size	Male/Female	Median age (year)	Type of study	Inhibitor	Neoadjuvant treatment
Bando et al., 2022 (18)	Non-China	44	29/15	59.5	Single-arm study	PD-1	Chemotherapy + LCRT + immunotherapy
Li et al., 2024 (19)	China	25	19/6	58	Single-arm study	PD-1	Chemoimmunotherapy + LCRT + chemotherapy
Lin et al., 2021 (20)	China	30	17/13	57	Single-arm study	PD-1	SCRT + Chemoimmunotherapy
Xiao et al., 2024 (21)	China	67	43/24	56	RCT	PD-1	Chemoimmunotherapy + LCRT
Shamseddine et al., 2020 (29)	Non-China	13	9/4	62	Single-arm study	PD-L1	SCRT+ Chemoimmunotherapy
Gao et al., 2023 (22)	China	26	14/12	60.5	Single-arm study	PD-1	LCRT + Chemoimmunotherapy
Lin et al., 2024 (23)	China	113	75/38	NA	RCT	PD-1	SCRT + Chemoimmunotherapy
George et al., 2022 (24)	Non-China	45	NA	NA	Single-arm study	PD-L1	Chemotherapy + LCRT + immunotherapy
Feng et al., 2024 (25)	China	22	NA	56	Single-arm study	PD-1	SCRT + Chemoimmunotherapy
Takahashi et al., 2023 (26)	Non-China	25	18/7	63	Single-arm study	PD-1	LCRT + Chemoimmunotherapy
Gooyer et al., 2024 (27)	Non-China	44	34/10	NA	Single-arm study	PD-L1	SCRT + Chemoimmunotherapy
Zhou et al., 2024 (28)	China	16	11/5	NA	RCT	PD-1	Chemoimmunotherapy
Xia et al., 2024 (30)	China	121	NA/NA	NA	RCT	PD-1	SCRT + Chemoimmunotherapy

### 3.2 Primary outcomes: pCR, MPR, cCR and anus preserving rate

**Figure 2:**
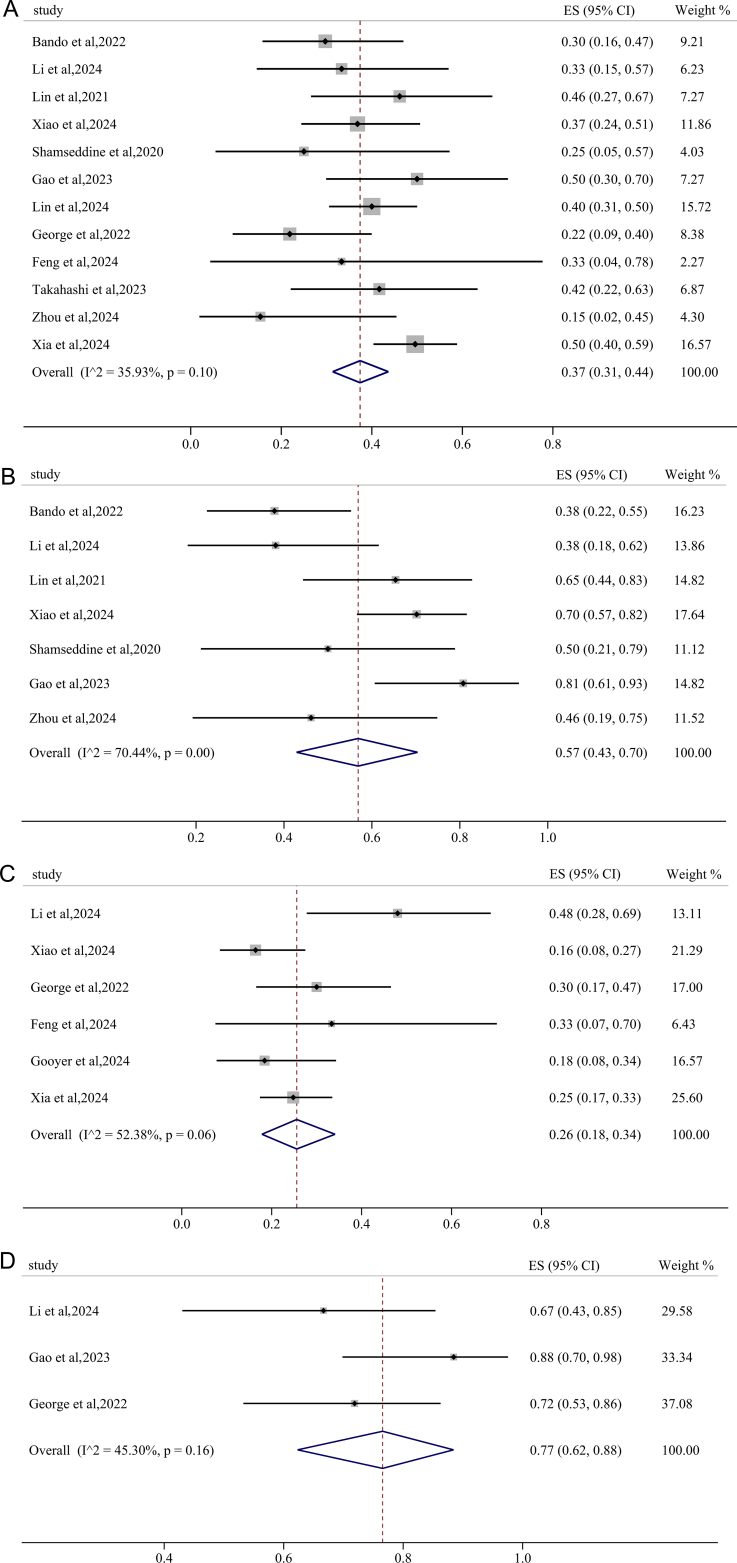
Primary outcomes of neoadjuvant immunotherapy for non-metastatic pMMR/MSS rectal cancer. **(A)** pathological complete response (pCR); **(B)** major pathological response (MPR); **(C)** clinical complete response (cCR); **(D)** anus preservation rate.

Twelve studies reported results of pCR and the pooled pCR rate was 37% (95%CI: 0.31, 0.44) with small heterogeneity (*I^2^* = 35.93%, p=0.10) (**Figure 2A**). Seven studies reported the clinical data on MPR, and the pooled MPR was 57% [(95%CI: 0.43, 0.70), *I^2^* = 70.44%, p=0.00] (**Figure 2B**). Six studies reported cCR, resulting in a pooled cCR rate of 26% (95%CI: 0.18, 0.34, *I^2^* = 52.38%, p=0.06) (**Figure 2C**). As illustrated in **Figure 2D**, three studies reported anus preservation rate with a pooled rate of 77% (95%CI: 0.62, 0.88, *I^2^* = 45.30%, p=0.16).

### 3.3 Safety: TRAEs and irAEs

**Figure 3:**
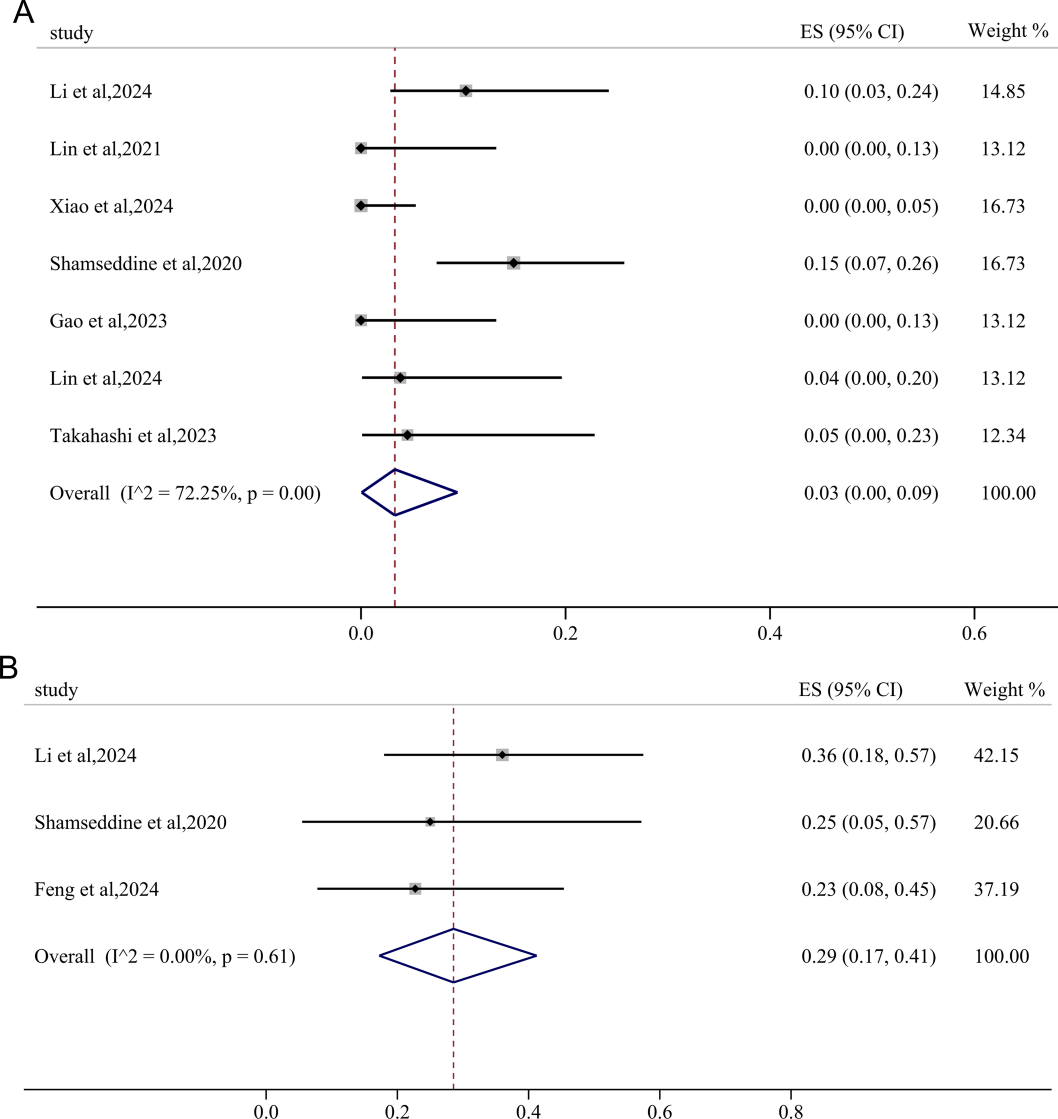
Forest plots showing the adverse effects of immunotherapy-based neoadjuvant therapy for RC. **(A)** immunotherapy -related adverse effects (irAEs)≥3 grades; **(B)** treatment-related adverse effects (TRAEs)≥3 grades.

The incidence of irAEs≥3 grades was extracted from seven studies, yielding a pooled rate was 3% (95%CI: 0.00, 0.09; *I^2^* = 72.25%, p=0.00) (**Figure 3A**). Using a fixed-effects model with low heterogeneity (*I^2^* = 0.00%, p=0.61), the pooled incidence of TRAEs≥3 grades was found to be 29% (95%CI: 0.17, 0.41) (**Figure 3B**).

### 3.4 Publication bias and influence analysis

**Figure 4:**
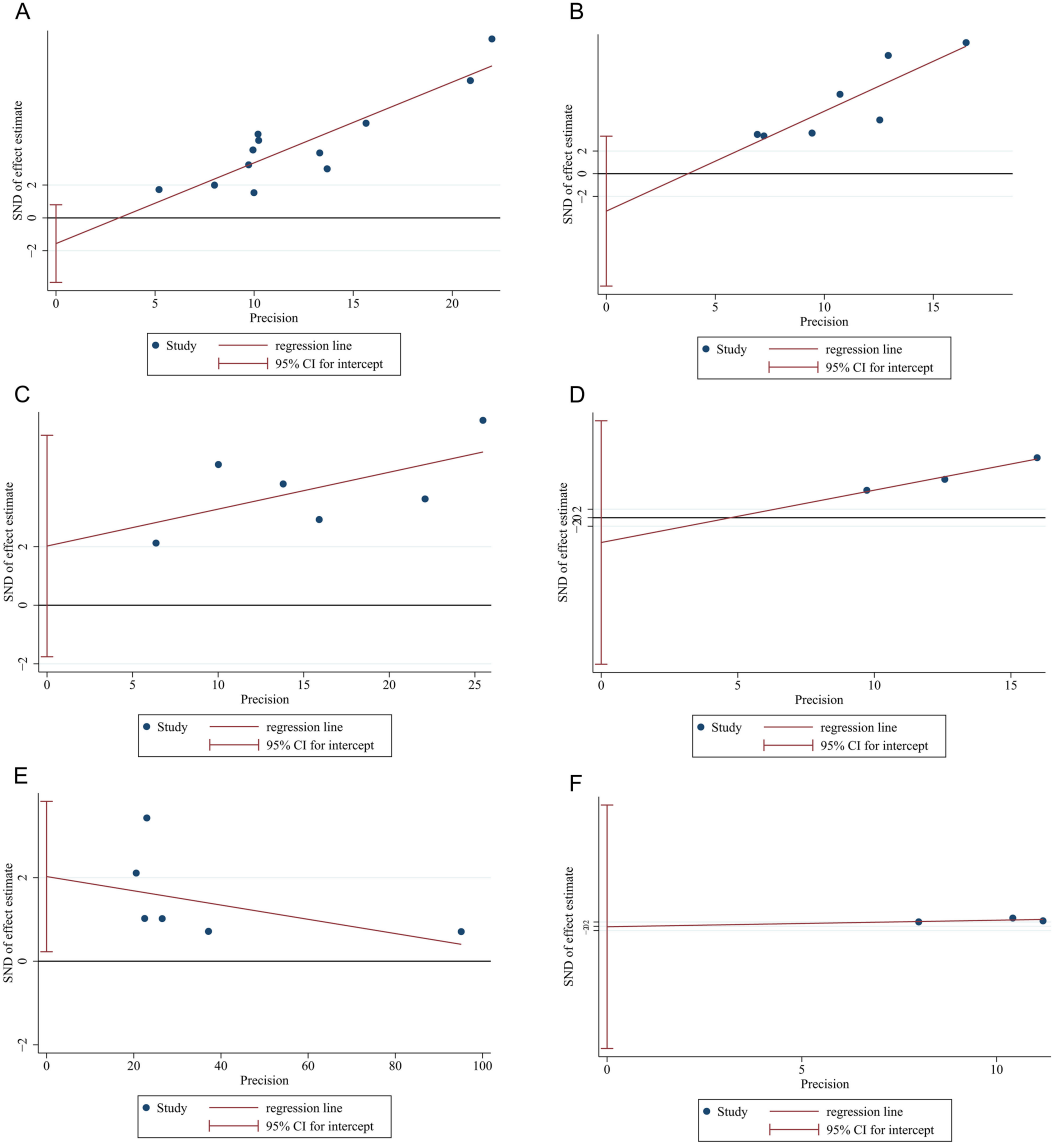
Funnel plots of Egger`s publication bias analysis for **(A)** pCR, **(B)** MPR, **(C)** cCR, **(D)** anus preservation rates, **(E)** incidence of irAE≥3 grades and **(F)** incidence of TRAEs ≥3 grades.

Funnel plots were employed to assess the potential for publication bias among the studies incorporated in the meta-analysis. As illustrated in **Supplementary Figure 1**, the funnel plots exhibited a certain degree of asymmetry, which may indicate possible publication bias stemming from a lack of RCT articles. Next, Egger’s and Begg’s tests were carried out to evaluate the publication bias. P value of Egger’s and Begg’s tests for pCR, MPR, cCR, anus preservation rate and TRAEs rates were all>0.05 (P>|t|=0.17, 0.26, 0.21, 0.24 and 0.97, respectively; Pr>|z|=1.70, 1.63, 0.26, 1.96 and 1.00, separately) and the symmetry of funnel plots from Egger`s publication bias analysis also suggested the stability of the results and the absence of bias and (**Supplementary File 2**, **Figures 4A–D, F**). Conversely, p value of Egger’s and Begg’s tests for irAEs ≥ 3 grades were both<0.05 (P>|t|=0.03, Pr>|z|=0.02) (**Supplementary File 2**) with associated funnel plots from Egger`s publication bias analysis exhibiting asymmetry (**Figure 4E**), indicating the publication bias existed in the study. Subsequently, the trim and fill method analysis was performed to evaluate the impact of publication bias. Based on the analysis results of the trim and fill method, there was almost minimal variation in the outcomes reinforcing the stability of the results (**Supplementary File 3**, **Supplementary Figure 2**). The significance remained consistent before and after the trim and fill method analysis, indicating that the combined effect size for the irAEs rate was not influenced by publication bias. Sensitivity analysis revealed that the exclusion of individual studies did not result in statistically significant changes in the combined analysis (**Supplementary Figure 3**), hereby suggesting that the overall conclusions drawn from this investigation can be regarded as valid and reliable.

### 3.5 Subgroup analysis

#### 3.5.1 Subgroup based on type of radiotherapy

**Figure 5:**
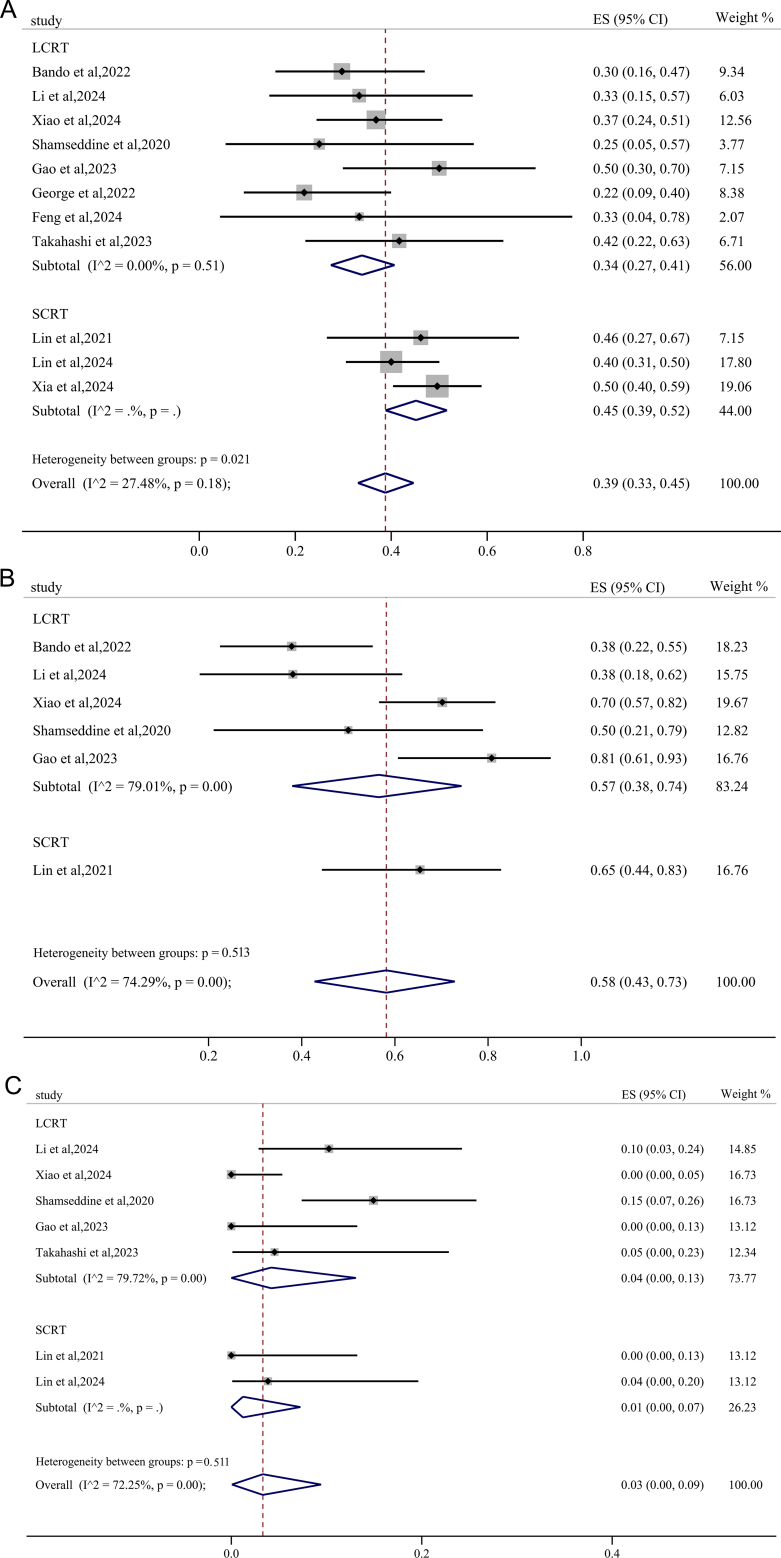
The forest figure of response rate (pCR, MPR and irAEs) based on radiotherapy strategies subgroup analysis. **(A)** pCR rate based on radiotherapy strategies subgroup analysis; **(B)** MPR rate based on radiotherapy strategies subgroup analysis; **(C)** irAEs rate based on radiotherapy strategies subgroup analysis.

The pooled pCR and MPR in the short course radiation therapy (SCRT) subgroup was 45% (95%CI: 0.39, 0.52) and 65% (95%CI: 0.44, 0.83), whereas the pooled pCR and MPR in the LCRT subgroup was 34% (95%CI: 0.27, 0.41) and 57% (95%CI: 0.38, 0.74), respectively (**Figures 5A, B**), all lower than the long course radiation therapy (LCRT) subgroup, especially the pooled pCR. However, the pooled incidence of irAEs≥3 grades in the LCRT subgroup (4.2%, 95%CI: 0.00, 0.13) exceeded that in the SCRT subgroup (1%, 95%CI: 0.00, 0.07) (**Figure 5C**).

#### 3.5.2 Subgroup based on PD-1/PD-L1 inhibitors

**Figure 6:**
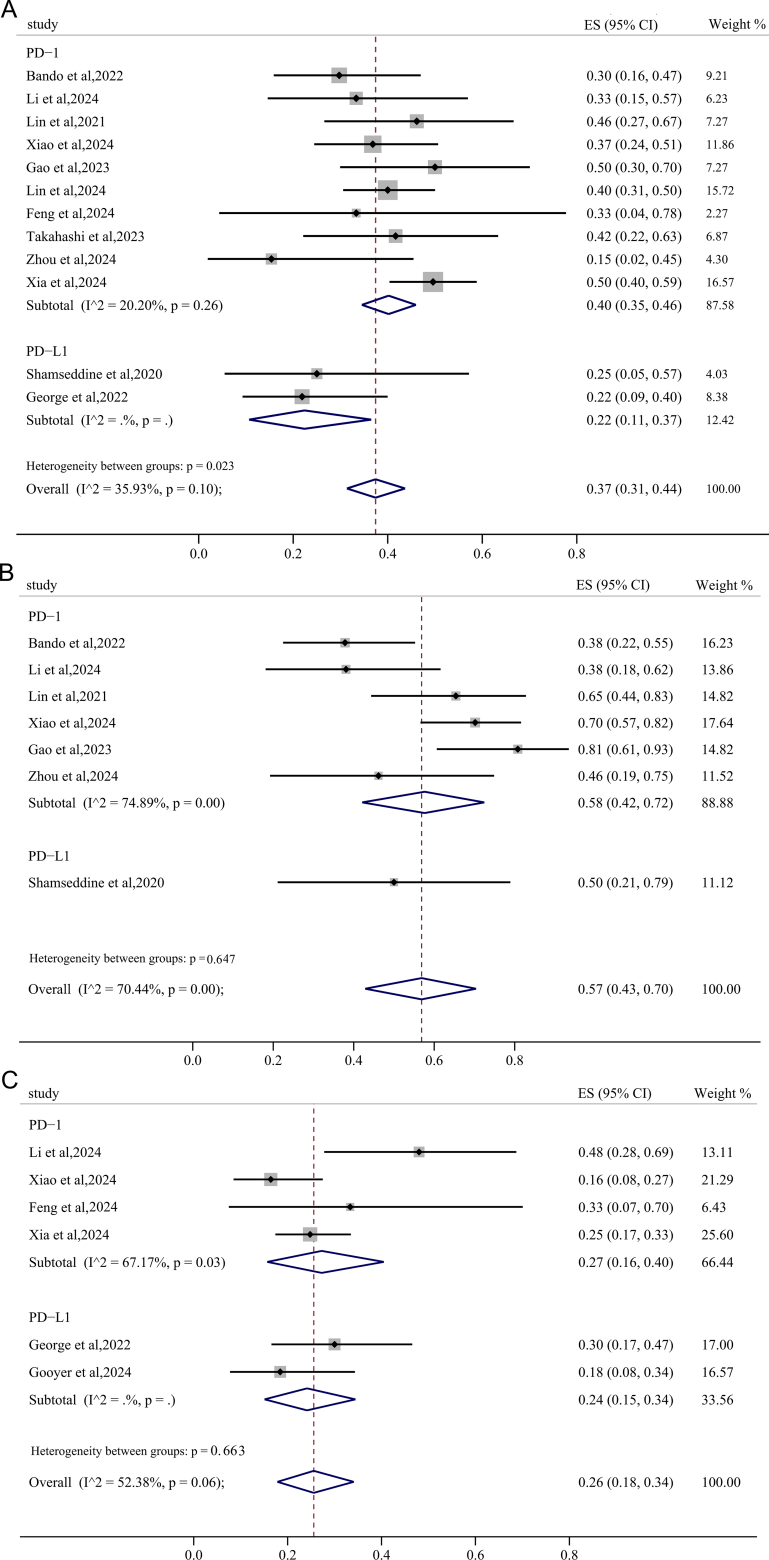
The forest figure of response rate (pCR, MPR, cCR) based on PD-1/PD-L1 inhibitors subgroup analysis. **(A)** pCR rate based on PD-1/PD-L1 inhibitors subgroup analysis; **(B)** MPR rate based on PD-1/PD-L1 inhibitors subgroup analysis; **(C)** cCR rate based on PD-1/PD-L1 inhibitors subgroup analysis.

The PD-1 subgroup exhibited a pooled pCR of 40% (95%CI: 0.35, 0.46), which was significantly higher than that observed in the PD-L1 subgroup (22%, 95%CI: 0.11, 0.37) (**Figure 6A**). Similarly, the pooled MPR and cCR in the PD-1 subgroup (58%, 95%CI: 0.42, 0.72; 27%, 95%CI: 0.16, 0.40) was higher than these in the PD-L1 subgroup (50.0%, 95%CI: 0.21, 0.79; 24%, 95%CI: 0.15, 0.34) (**Figures 6B, C**), though there were no significant declines in heterogeneity.

#### 3.5.3 Subgroup based on treatment sequence

**Figure 7:**
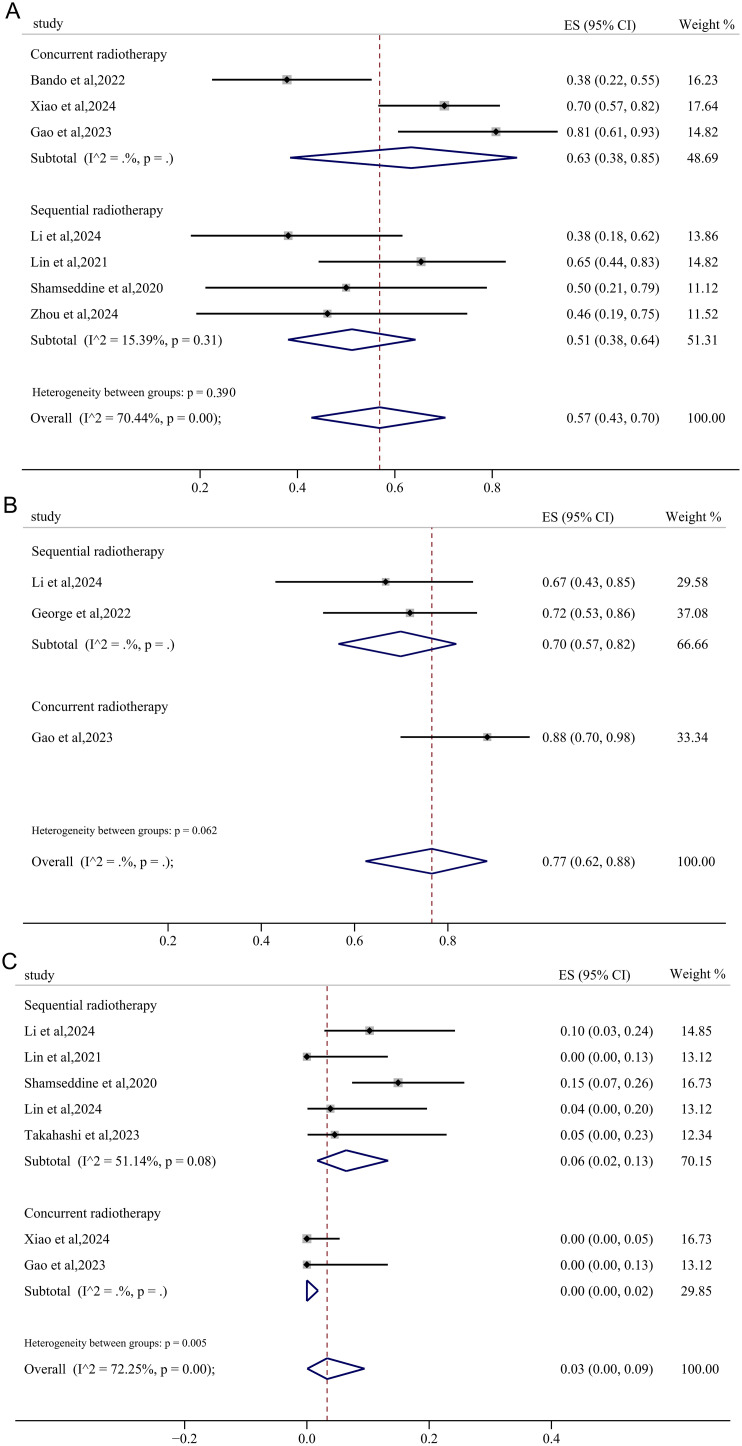
The forest figure based on treatment sequence subgroup analysis. The forest figure of MPR **(A)**, anus preservation rate **(B)** and incidence of irAEs ≥ 3 grades **(C)** based on sequence of immunotherapy and radiotherapy subgroup analysis.

Subsequent subgroup analyses were conducted to evaluate the sequence of immunotherapy and radiotherapy administration in the neoadjuvant context. The pMMR/MSS RC cohorts receiving concurrent immunotherapy and radiotherapy demonstrated a pooled MPR of 63% (95%CI: 0.38, 0.85) and an anal preservation rate of 88% (95%CI: 0.70, 0.99), respectively, both exceeding the outcomes observed in those undergoing sequential administration (**Figures 7A, B**). Furthermore, a notable reduction in heterogeneity for MPR was observed. Though there was only minimal declination in heterogeneity for the rate of irAEs, the pooled incidence of irAEs≥3 grades in the sequential radiotherapy group was significant higher compared to the concurrent group (6% vs 0.0%) (**Figure 7C**).

#### 3.5.4 Subgroup analysis based on clinical T and N category

**Figure 8:**
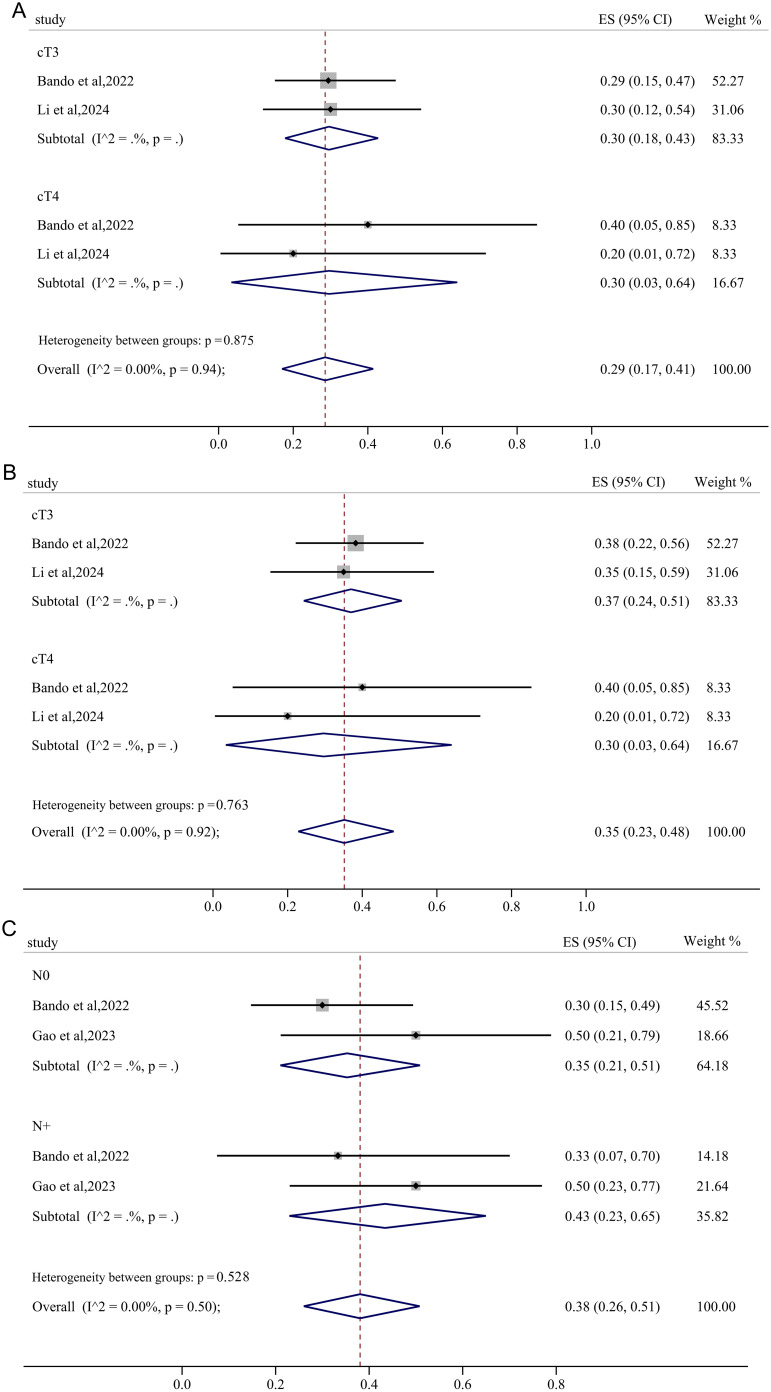
The forest figure based on clinical T category and N subgroup analysis. **(A)** pCR rate on clinical T category subgroup analysis; **(B)** MPR rate on clinical T category subgroup analysis; **(C)** pCR rate on clinical N category subgroup analysis.

In the subgroup analysis stratified by clinical T category, both the cT3 and cT4 subgroups exhibited a pooled pCR of 30% with negligible heterogeneity (**Figure 8A**). With negligible heterogeneity, the pooled MPR in cT3 and cT4 group was 37% (95%CI: 0.24, 0.51) and 30% (95%CI: 0.03, 0.64), respectively, demonstrating that the subgroup analysis based on clinical T category significantly diminished the heterogeneity in MPR (**Figure 8B**). Interestingly, for subgroup analysis stratified by clinical N category, pMMR/MSS non-metastatic RC patients with lymph node metastasis achieved a pooled pCR of 43% (95%CI: 0.23, 0.65) after receiving immunotherapy-based neoadjuvant treatment, higher than the pooled pCR of 35% (95%CI: 0.21, 0.51) in RC patients without lymph node metastasis (**Figure 8C**).

## 4 Discussion

In 2015, researchers from Johns Hopkins Hospital initially disclosed the KEYNOTE-016 study at the ASCO Annual Meeting, identifying MSI-H or dMMR as molecular markers indicative of immunotherapy responsiveness in metastatic CRC, thus heralding a transformative era in CRC immunotherapy (31). The NICHE study encompassing both dMMR and pMMR early-stage colon cancer patients first explores the efficacy and safety of NIT. In the primary results, MPR and pCR of the 20 dMMR patients are 95% and 60%, respectively, while the MPR rate is 20% in patients with pMMR colon cancer, which opens the door of NIT for CRC (32).

This systematic review comprehensively analyzed data from the 13 studies to assess the efficacy and safety of NIT in non-metastatic pMMR/MSS RC patients, revealing favorable outcomes with pooled pCR, MPR, cCR and anus preserving rate of 37%, 57%, 26% and 77%, respectively. Moreover, NIT did not significantly elevate the incidence of AEs, with the pooled rate of irAEs and TRAEs≥3 grades being 3% and 29% separately. Therefore, the implementation of immunotherapy in the neoadjuvant settings in patients with non-metastatic pMMR/MSS RC is a promising therapeutic strategy.

To evaluate the publication bias, Egger’s and Begg’s tests and funnel plots were employed. The asymmetry of funnel plots for irAEs indicated the possibility of publication bias, and the p value of the next Egger’s and Begg’s tests for irAEs ≥ 3 grades all<0.05 also suggested the potential of publication bias (**Figure 4E**, **Supplementary File 2**), which may be caused by lack of RCTs, small sample size, incomplete or selective reports due to the fact that the exploration of NIT for RC is still in its nascent stages. The existing of publication bias may exaggerate treatment effects, mislead clinical practice and decision-making and affect the generality and reliability of results. However, the consistent significance before and after the trim and fill method analysis, indicating that the combined effect size for the irAEs rate in our study was not influenced by publication bias and the conclusions of our analysis (**Supplementary File 3**, **Supplementary Figure 2**).

Given the potential heterogeneity and publication bias, subgroup analyses were conducted. Significant reductions in heterogeneity were observed in the pooled pCR rates based on the type of radiotherapy and inhibitor subgroup analysis. Subsequent subgroup analysis based on the sequence of immunotherapy and radiotherapy application demonstrated a substantial decrease in heterogeneity in pooled pCR and MPR, suggesting that the treatment sequence of immunotherapy and radiotherapy in different studies may contribute to the heterogeneity in pCR and MPR outcomes. Similarly, subgroup analysis based on the clinical T category also revealed a reduction in heterogeneity of pCR and MPR, indicating another potential source of heterogeneity in the pooled pCR and MPR results. However, Egger’s and Begg’s tests yielded p values >0.05 for pCR, MPR, cCR, anus preservation rates and TRAEs, indicating the absence of publication bias. Sensitivity analysis further confirmed the stability of the pooled pCR. Although the p values of Egger’s and Begg’s tests for the incidence of irAEs of grade ≥3 were both <0.05, the consistent significance before and after the trim and fill method demonstrated that the combined effect size of irAEs rates was not influenced by the potential existed publication bias, ensuring the robustness of the findings.

Numerous studies indicated that a higher pCR is associated with favorable prognosis (30, 33). As one of the standard preoperative treatment for LARC, TNT showed superior rates of pCR compared with conventional CRT (29.9% versus 14.9%), as well as a reduction in distant relapse in meta-analysis of Anup Kasi, MD et al. (34). However, the relatively low pCR rates and high rates of distant metastasis and local disease recurrence following TNT and conventional CRT remain challenges in the management of LARC. In our meta-analysis, a pooled pCR rate of 37% was observed in patients with pMMR/MSS rectal cancer receiving NIT, surpassing the rates achieved with TNT and conventional CRT.

For patients with early-stage and LARC, surgery still plays a critical role in the treatment course. However, older patients with multiple comorbidities may face a heightened risk of mortality and severe complications post-surgery, rendering them unsuitable candidates for operative intervention. Although advancements in medical technology have significantly enhanced anal preservation rates in RC patients, the rectum and anus preservation remain challenging for individuals with ultra-low rectal cancer (35). Therefore, in addition to tumor burden reduction and survival improvement, organ preservation is a critical consideration in RC treatment.

The concept of the “wait-and-see” strategy for RC patients achieving cCR after neoadjuvant therapy, initially proposed by Prof. Habr-Gama from Brazil in 2004, has garnered increasing attention due to its positive impact on quality of life (QoL) and minimal effect on long-term survival outcomes (6). In 2016, Martens et al. reported a cCR rate of 17% among patients with RC who underwent neoadjuvant CRT (36). A meta-analysis evolving seventeen studies revealed a pooled cCR rate of 22.4% following conventional neoadjuvant chemoradiotherapy treatment (37). In our meta-analysis the pooled cCR rate of 26%, higher than the previous clinical study and meta-analysis, supported the utilization of NIT in patients with non-metastatic MSS RC and provided new options, particularly for older patients with comorbidities or those averse to surgery.

TRAEs or irAEs, which refers to a multitude of systems, are unignorable problems in immunotherapy-based neoadjuvant treatment. Previous studies have reported that grades 3−5 TRAEs and irAEs were observed in 33% and 19% of advanced CRC patients receiving NIT, respectively (38). The pooled TRAEs rate of 29% and irAEs rate of 3% in our study demonstrated the favorable tolerability of NIT in non-metastatic pMMR/MSS RC. The satisfactory pooled pCR, MPR, cCR, anus preservation rate and low rate of AEs≥3 grades further support the use of NIT in non-metastatic pMMR/MSS RC patients. While the inconsistency in the identification of pCR and cCR warrants more future studies to solve the problem.

In the multicenter, open-label, randomized, controlled, phase III RAPIDO trial, patients in the experiment group receiving SCRT in neoadjuvant treatment period showed higher treatment compliance, reduced risk of disease recurrence and metastasis than the standard of care group receiving LCRT (39). Similarly, in the UNION trials, LARC patients received SCRT followed by chemoimmunotherapy in the neoadjuvant period achieved higher pCR rate with well-tolerated safety profile than those treated with LCRT in neoadjuvant period (23). Preclinical studies also indicated that SCRT enhanced the infiltration of tumor-specific CD8+ T cells in draining lymph nodes, leading to improved local and distant anti-tumor effects compared to conventional fractionation (40). In concordance with the previous findings, the pooled pCR and MPR rates in the SCRT subgroup were 45.2% and 65.4%, respectively, both higher than the LCRT groups. The incidence of irAEs in the SCRT subgroup was 1%, significantly lower than the LCRT group. The favorable characteristics of SCRT treatment, including low toxicity, positive therapeutic effects, cost-effectiveness, and convenience, have garnered increasing interest in clinical practice.

PD-1 and PD-L1 inhibitors demonstrated comparable survival outcomes and safety profiles in patients with solid tumors (41, 42). However, non-metastatic pMMR/MSS RC patients receiving PD-1 inhibitors in the neoadjuvant treatment period in our subgroup analysis had a higher pCR rate and slightly higher or comparable MPR or cCR compared with patients receiving PD-L1 inhibitors. In addition, anus preservation rate in RC patients receiving PD-1 inhibitors in the neoadjuvant treatment period was also slightly higher than those received PD-L1 inhibitors (**Supplementary Figure 4**), further supporting the clinical utility of NIT for non-metastatic pMMR/MSS RC patients.

Discrepancies remain regarding the sequencing of radiotherapy and chemotherapy/immunotherapy in oncological treatment. In patients diagnosed with stage III non-small cell lung cancer, concurrent radiotherapy combined with chemotherapy demonstrated enhanced survival benefits but with increased toxicity compared to sequential radiotherapy (43). Conversely, another investigation revealed that concurrent radiotherapy paired with immunotherapy resulted in superior survival rates with reduced toxicity relative to sequential radiotherapy (44). In our analysis, RC patients undergoing concurrent radiotherapy alongside immunotherapy during the neoadjuvant phase achieved a MPR and anal preservation rates of 63% and 88%, respectively, and both of which surpassed the sequential radiotherapy group. Besides, the incidence of irAEs≥3 grades in the concurrent radiotherapy group was significantly lower than the sequential radiotherapy group. Prior studies indicated that concurrent radiotherapy plus camrelizumab elevated the expression levels of activation molecules CD38 and HLA-DR on CD8+ T cells, thereby enhancing the cytotoxicity and activation of PD-1+CD8+ T cells, which correlated with improved prognosis in patients with esophageal squamous cell carcinoma (45), potentially elucidating our findings. Nonetheless, additional research is warranted to further investigate these outcomes.

Clinical T4 stage, N2 stage, and EMVI positivity are recognized as high-risk factors for RC patients, typically associated with unfavorable prognoses. Nevertheless, the UNION trials (23) and numerous other clinical studies evolving RC patients with T3, T4 or N2 stage, demonstrate satisfactory survival outcomes and well tolerance when treated with specific strategies. Our subgroup analysis also revealed that patients with T4 RC exhibited a comparable pCR rate to those classified as clinical T3, while patients with lymph node metastasis demonstrated an even higher pCR rate compared to those without such metastasis. Therefore, for non-metastatic MSS RC patients with T4 category or other high-risk factors, the NIT may play a pivotal role.

In the KEYNOTE-966 study, patients with biliary tract cancers receiving pembrolizumab achieve a mOS of 14.1 (95%CI: 10.4-17.7) months, significantly exceeding the outcomes observed in the global cohort (46). The KEYNOTE-181 study also reveals that immunotherapy treatment brings significant OS benefit and better prognosis for Chinese population compared with the whole population (47). Currently, there is a dearth of studies assessing the efficacy and safety of NIT in both Chinese and non-Asian patients with MSS RC. Our subgroup analysis revealed that the pooled pCR, MPR and cCR rates in the Chinese cohort were 41%, 65% and 27% respectively, superior to those in non-Chinese populations (**Supplementary Figure 5**). Although approximately 61.5% of the trials included in our analysis were conducted within Chinese populations, publication bias assessments affirmed the robustness of the findings. Thus, our study offers valuable insights and support for future research and clinical applications when addressing patients from diverse ethnic backgrounds.

This systematic review acknowledges certain limitations. Firstly, while 4 RCTs were incorporated, the majority of the included studies were single-arm, phase II prospective trials or conference abstracts, leading to a limited patient cohort and incomplete clinical data. Secondly, the application of NIT for RC is still in its nascent stages, with most studies primarily reporting initial findings and lacking long-term survival data. Therefore, further multi-center, large-sample clinical trials were conducted to improve the reliability and universality of the study results and evaluate the long-term survival outcomes. Additionally, the small sample sizes and heterogeneity in treatment regimens and follow-up durations may also impact the results of the study, which need to be explored with more future studies. To optimize the treatment strategy for RC, the efficacy and safety of NIT combined with other therapeutic modalities need to be further investigated. Besides, by identifying molecular markers associated with the response of NIT, precise stratification of patients can be achieved to provide a basis for individualized treatment.

## 5 Conclusion

Our study has synthesized and examined the latest trials concerning neoadjuvant immunotherapy for non-metastatic pMMR/MSS RC patients, analyzing various outcomes. Due to relatively small sample size, heterogeneity between studies and uneven levels of included studies, there were no statistical significance for MPR in subgroup analysis based on radiotherapy type, PD-1/PD-L1 inhibitors and other factors, but statistically significant pCR rate based on radiotherapy strategies, PD-1/PD-L1 inhibitors subgroup analysis and other satisfactory outcomes in the subgroup analysis indicates that NIT is promising for the treatment of pMMR/MSS RC patients with an acceptable safety profile. Moreover, the high response rates among MSS patients, satisfactory anal preservation rates, and low incidences of TRAEs and irAEs provide a reference for future research and clinical practice. However, in light of these limitations, there is an urgent need for large-scale, randomized controlled trials focusing on neoadjuvant approaches for non-metastatic MSS RC.

## Data Availability

The original contributions presented in the study are included in the article/**Supplementary Material**. Further inquiries can be directed to the corresponding author.
