# Supplementary material for: Neoadjuvant immunotherapy for DNA mismatch repair proficient/microsatellite stable non-metastatic rectal cancer: a systematic review and meta-analysis
Source: Front Immunol. 2025 Jan 27;16:1523455. doi: 10.3389/fimmu.2025.1523455 (PMC11808008; doi:10.3389/fimmu.2025.1523455)

**1.Egger and Begg test for pCR**

**Egger test**


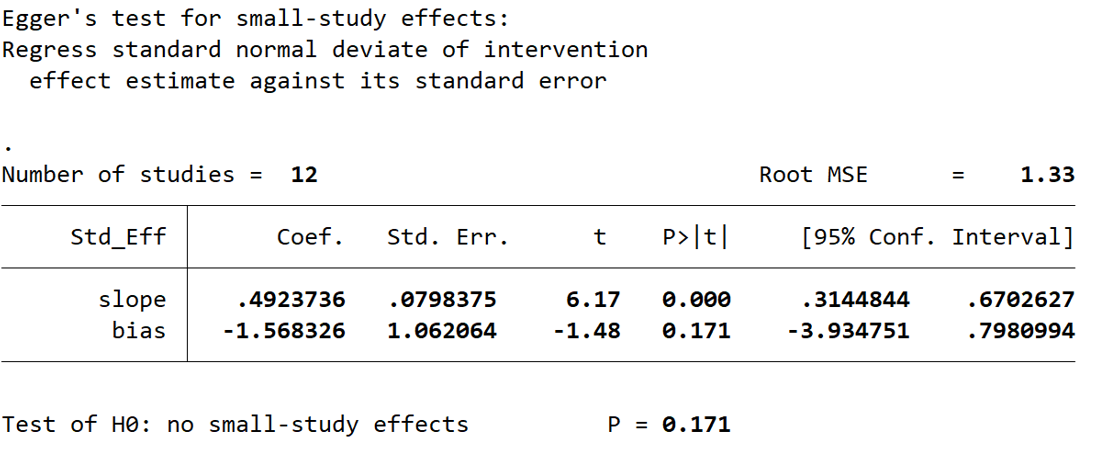


**Begg test**


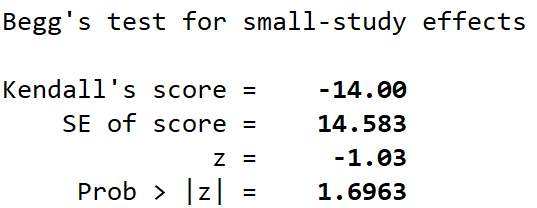


**2.Egger and Begg test for MPR**

**Egger test**


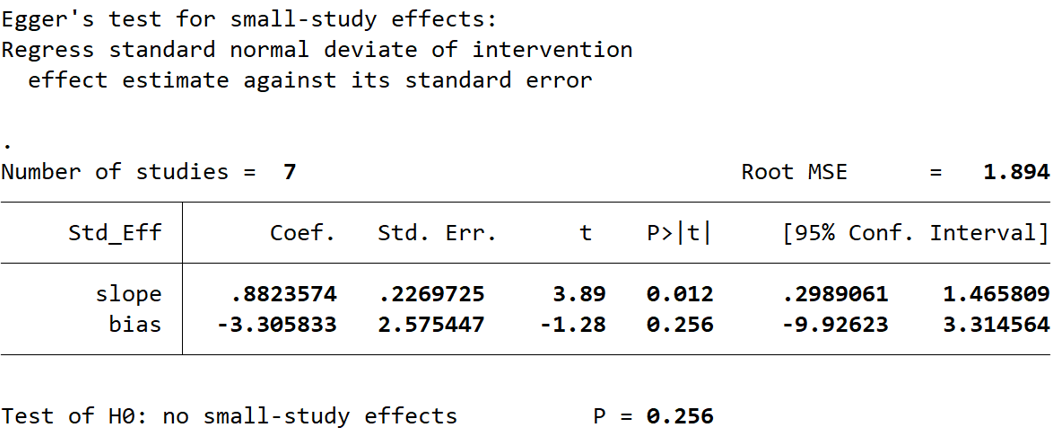


**Begg test**


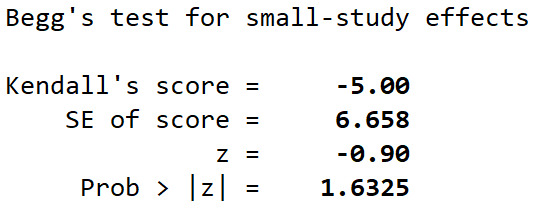


**3.Egger and Begg test for cCR**

**Egger test**


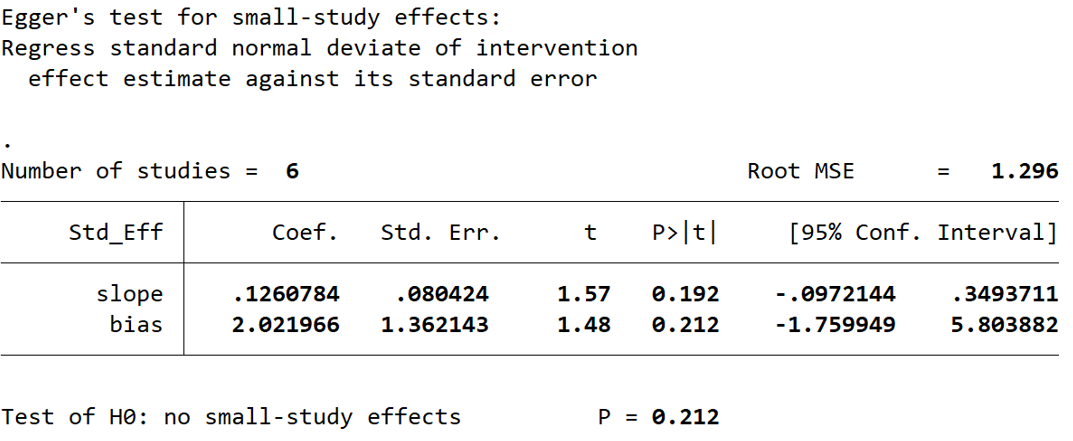


**Begg test**


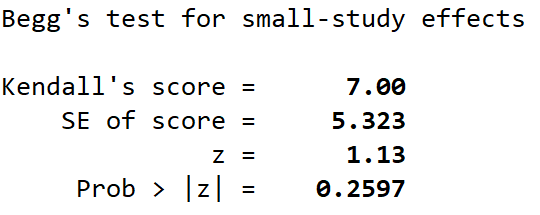


**4.Egger and Begg test for anus preservation rate**

**Egger test**


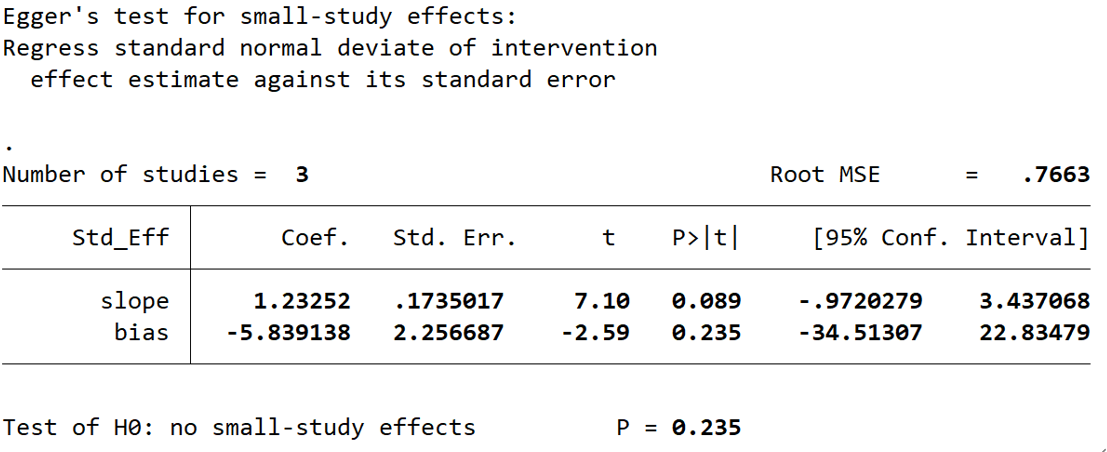


**Begg test**


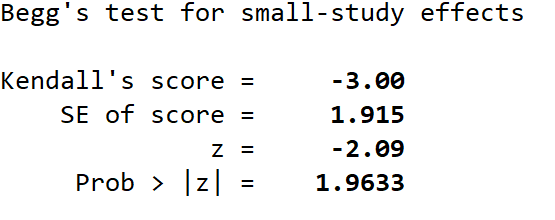


**5.Egger and Begg test for ≥ 3 grades irAEs rates**

**Egger test**


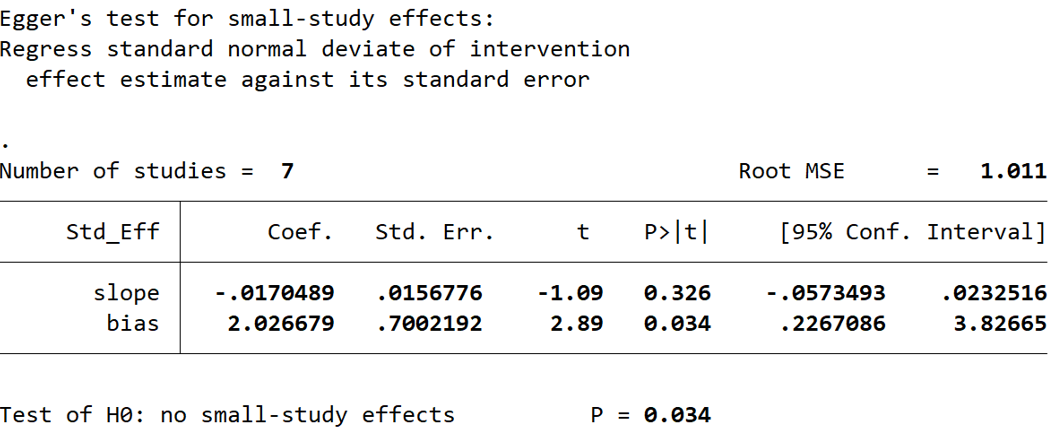


**Begg test**


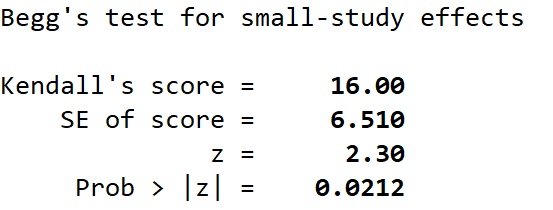


**6.Egger and Begg test for ≥ 3 grades TRAEs rates**

**Egger test**


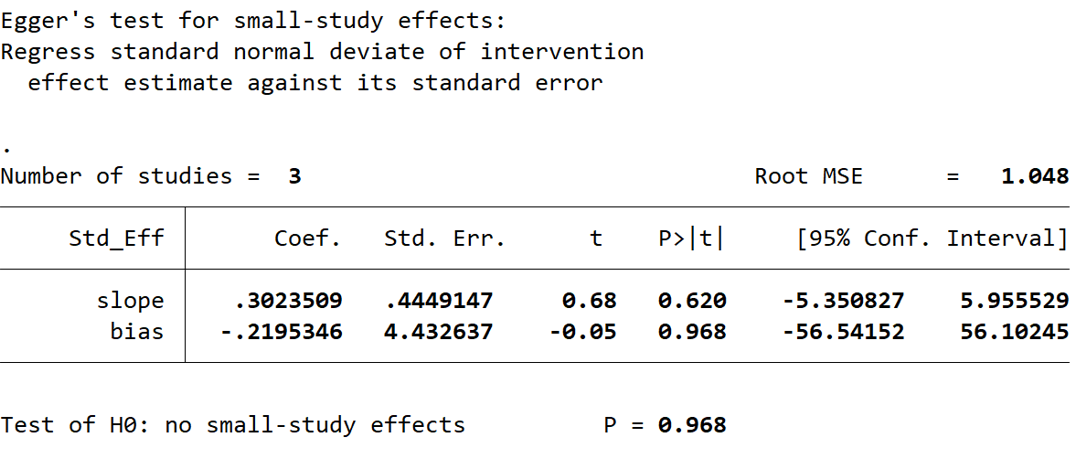


**Begg test**


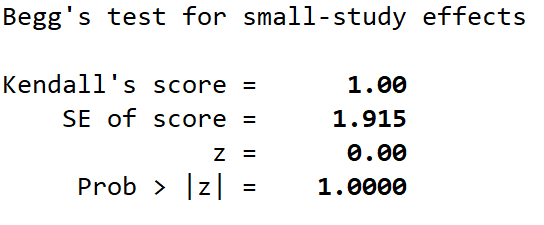

Supplement: Supplementary file 1 [file DataSheet1.docx]
