# Supplementary material for: Neoadjuvant immunotherapy for DNA mismatch repair proficient/microsatellite stable non-metastatic rectal cancer: a systematic review and meta-analysis
Source: Front Immunol. 2025 Jan 27;16:1523455. doi: 10.3389/fimmu.2025.1523455 (PMC11808008; doi:10.3389/fimmu.2025.1523455)
Supplement: Supplementary file 2 [file DataSheet2.docx]

**Supplimentary file 1 Search strategy**

**Pubmed**

| No. | Search Details | Results |
| --- | --- | --- |
| 14 | ("immunotherapy"[MeSH Terms] OR "immunotherapy"[All Fields] OR "immunotherapies"[All Fields] OR "immunotherapy s"[All Fields]) AND ("colorectal neoplasms"[MeSH Terms] OR ("colorectal"[All Fields] AND "neoplasms"[All Fields]) OR "colorectal neoplasms"[All Fields] OR ("colorectal"[All Fields] AND "cancer"[All Fields]) OR "colorectal cancer"[All Fields]) | 8,451 |
| 13 | ("Rectal Neoplasms"[MeSH Terms] OR ("Rectal Neoplasms"[Title/Abstract] OR "neoplasm rectal"[Title/Abstract] OR "rectal neoplasm"[Title/Abstract] OR "neoplasms rectal"[Title/Abstract] OR "rectum neoplasms"[Title/Abstract] OR "neoplasm rectum"[Title/Abstract] OR "rectum neoplasm"[Title/Abstract] OR "rectal tumors"[Title/Abstract] OR "rectal tumor"[Title/Abstract] OR "tumor rectal"[Title/Abstract] OR "cancer of rectum"[Title/Abstract] OR "rectum cancers"[Title/Abstract] OR "cancer of the rectum"[Title/Abstract] OR "rectal cancer"[Title/Abstract] OR "cancer rectal"[Title/Abstract] OR "rectal cancers"[Title/Abstract] OR "rectum cancer"[Title/Abstract] OR "cancer rectum"[Title/Abstract] OR "colorectal cancer"[Title/Abstract] OR "colorectal neoplasms"[Title/Abstract])) AND ("Immunotherapy"[MeSH Terms] OR ("Immunotherapy"[Title/Abstract] OR "immunotherapies"[Title/Abstract] OR "immunotherapeutic"[Title/Abstract] OR "immunotherapeutic agents"[Title/Abstract] OR "checkpoint inhibitor"[Title/Abstract] OR "checkpoint inhibitors"[Title/Abstract] OR "immune checkpoint inhibitors"[Title/Abstract] OR "checkpoint blockade"[Title/Abstract] OR "checkpoint blockades"[Title/Abstract] OR "programmed cell death protein 1"[Title/Abstract] OR "programmed cell death 1"[Title/Abstract] OR "programmed death 1"[Title/Abstract] OR "programmed death protein 1"[Title/Abstract] OR "programmed cell death receptor 1 inhibitor"[Title/Abstract] OR "programmed cell death receptor 1 inhibitors"[Title/Abstract] OR "programmed cell death 1 receptor inhibitor"[Title/Abstract] OR "programmed cell death 1 receptor inhibitors"[Title/Abstract] OR "anti pd 1 antibody"[Title/Abstract] OR "anti pd 1 antibodies"[Title/Abstract] OR "programmed cell death 1 ligand 1"[Title/Abstract] OR "programmed death 1 ligand 1"[Title/Abstract] OR "programmed death ligand 1"[Title/Abstract] OR "PD-1"[Title/Abstract] OR "PD1"[Title/Abstract] OR "PD-L1"[Title/Abstract] OR "PDL1"[Title/Abstract] OR "anti pd l1 antibody"[Title/Abstract] OR "anti pd l1 antibodies"[Title/Abstract] OR "anti pd l1 inhibitor"[Title/Abstract] OR "anti pd l1 inhibitors"[Title/Abstract] OR "pd 1 inhibitors"[Title/Abstract] OR "pd l1 inhibitors"[Title/Abstract] OR "ctla 4 inhibitors"[Title/Abstract] OR "lag 3 inhibitors"[Title/Abstract] OR "tim 3 inhibitors"[Title/Abstract] OR "Pembrolizumab"[Title/Abstract] OR "Nivolumab"[Title/Abstract] OR "Tislelizumab"[Title/Abstract] OR "Ipilimumab"[Title/Abstract] OR "Envafolimab"[Title/Abstract])) AND ("Neoadjuvant Therapy"[MeSH Terms] OR ("neoadjuvant"[Title/Abstract] OR "preoperative"[Title/Abstract] OR "pre-operative"[Title/Abstract] OR "preoperation"[Title/Abstract] OR "preoperations"[Title/Abstract] OR "pre-operation"[Title/Abstract] OR "pre-operations"[Title/Abstract] OR "presurgical"[Title/Abstract] OR "pre-surgical"[Title/Abstract] OR "presurgery"[Title/Abstract] OR "pre-surgery"[Title/Abstract] OR "preresection"[Title/Abstract] OR "pre-resection"[Title/Abstract] OR "preresectional"[Title/Abstract] OR "before surgery"[Title/Abstract] OR "before resection"[Title/Abstract] OR "before operation"[Title/Abstract] OR "prior to resection"[Title/Abstract] OR "prior to surgery"[Title/Abstract] OR "prior to operation"[Title/Abstract])) | 468 |
| 12 | "Neoadjuvant Therapy"[MeSH Terms] OR "neoadjuvant"[Title/Abstract] OR "preoperative"[Title/Abstract] OR "pre-operative"[Title/Abstract] OR "preoperation"[Title/Abstract] OR "preoperations"[Title/Abstract] OR "pre-operation"[Title/Abstract] OR "pre-operations"[Title/Abstract] OR "presurgical"[Title/Abstract] OR "pre-surgical"[Title/Abstract] OR "presurgery"[Title/Abstract] OR "pre-surgery"[Title/Abstract] OR "preresection"[Title/Abstract] OR "pre-resection"[Title/Abstract] OR "preresectional"[Title/Abstract] OR "before surgery"[Title/Abstract] OR "before resection"[Title/Abstract] OR "before operation"[Title/Abstract] OR "prior to resection"[Title/Abstract] OR "prior to surgery"[Title/Abstract] OR "prior to operation"[Title/Abstract] | 470,047 |
| 11 | "neoadjuvant"[Title/Abstract] OR "preoperative"[Title/Abstract] OR "pre-operative"[Title/Abstract] OR "preoperation"[Title/Abstract] OR "preoperations"[Title/Abstract] OR "pre-operation"[Title/Abstract] OR "pre-operations"[Title/Abstract] OR "presurgical"[Title/Abstract] OR "pre-surgical"[Title/Abstract] OR "presurgery"[Title/Abstract] OR "pre-surgery"[Title/Abstract] OR "preresection"[Title/Abstract] OR "pre-resection"[Title/Abstract] OR "preresectional"[Title/Abstract] OR "before surgery"[Title/Abstract] OR "before resection"[Title/Abstract] OR "before operation"[Title/Abstract] OR "prior to resection"[Title/Abstract] OR "prior to surgery"[Title/Abstract] OR "prior to operation"[Title/Abstract] | 465,035 |
| 10 | "Neoadjuvant Therapy"[MeSH Terms] | 30,822 |
| 9 | "Immunotherapy"[MeSH Terms] OR "Immunotherapy"[Title/Abstract] OR "immunotherapies"[Title/Abstract] OR "immunotherapeutic"[Title/Abstract] OR "immunotherapeutic agents"[Title/Abstract] OR "checkpoint inhibitor"[Title/Abstract] OR "checkpoint inhibitors"[Title/Abstract] OR "immune checkpoint inhibitors"[Title/Abstract] OR "checkpoint blockade"[Title/Abstract] OR "checkpoint blockades"[Title/Abstract] OR "programmed cell death protein 1"[Title/Abstract] OR "programmed cell death 1"[Title/Abstract] OR "programmed death 1"[Title/Abstract] OR "programmed death protein 1"[Title/Abstract] OR "programmed cell death receptor 1 inhibitor"[Title/Abstract] OR "programmed cell death receptor 1 inhibitors"[Title/Abstract] OR "programmed cell death 1 receptor inhibitor"[Title/Abstract] OR "programmed cell death 1 receptor inhibitors"[Title/Abstract] OR "anti pd 1 antibody"[Title/Abstract] OR "anti pd 1 antibodies"[Title/Abstract] OR "programmed cell death 1 ligand 1"[Title/Abstract] OR "programmed death 1 ligand 1"[Title/Abstract] OR "programmed death ligand 1"[Title/Abstract] OR "PD-1"[Title/Abstract] OR "PD1"[Title/Abstract] OR "PD-L1"[Title/Abstract] OR "PDL1"[Title/Abstract] OR "anti pd l1 antibody"[Title/Abstract] OR "anti pd l1 antibodies"[Title/Abstract] OR "anti pd l1 inhibitor"[Title/Abstract] OR "anti pd l1 inhibitors"[Title/Abstract] OR "pd 1 inhibitors"[Title/Abstract] OR "pd l1 inhibitors"[Title/Abstract] OR "ctla 4 inhibitors"[Title/Abstract] OR "lag 3 inhibitors"[Title/Abstract] OR "tim 3 inhibitors"[Title/Abstract] OR "Pembrolizumab"[Title/Abstract] OR "Nivolumab"[Title/Abstract] OR "Tislelizumab"[Title/Abstract] OR "Ipilimumab"[Title/Abstract] OR "Envafolimab"[Title/Abstract] | 487,955 |
| 8 | "immunotherapy"[Title/Abstract] OR "immunotherapies"[Title/Abstract] OR "immunotherapeutic"[Title/Abstract] OR "immunotherapeutic agents"[Title/Abstract] OR "checkpoint inhibitor"[Title/Abstract] OR "checkpoint inhibitors"[Title/Abstract] OR "immune checkpoint inhibitors"[Title/Abstract] OR "checkpoint blockade"[Title/Abstract] OR "checkpoint blockades"[Title/Abstract] OR "programmed cell death protein 1"[Title/Abstract] OR "programmed cell death 1"[Title/Abstract] OR "programmed death 1"[Title/Abstract] OR "programmed death protein 1"[Title/Abstract] OR "programmed cell death receptor 1 inhibitor"[Title/Abstract] OR "programmed cell death receptor 1 inhibitors"[Title/Abstract] OR "programmed cell death 1 receptor inhibitor"[Title/Abstract] OR "programmed cell death 1 receptor inhibitors"[Title/Abstract] OR "anti pd 1 antibody"[Title/Abstract] OR "anti pd 1 antibodies"[Title/Abstract] OR "programmed cell death 1 ligand 1"[Title/Abstract] OR "programmed death 1 ligand 1"[Title/Abstract] OR "programmed death ligand 1"[Title/Abstract] OR "PD-1"[Title/Abstract] OR "PD1"[Title/Abstract] OR "PD-L1"[Title/Abstract] OR "PDL1"[Title/Abstract] OR "anti pd l1 antibody"[Title/Abstract] OR "anti pd l1 antibodies"[Title/Abstract] OR "anti pd l1 inhibitor"[Title/Abstract] OR "anti pd l1 inhibitors"[Title/Abstract] OR "pd 1 inhibitors"[Title/Abstract] OR "pd l1 inhibitors"[Title/Abstract] OR "ctla 4 inhibitors"[Title/Abstract] OR "lag 3 inhibitors"[Title/Abstract] OR "tim 3 inhibitors"[Title/Abstract] OR "Pembrolizumab"[Title/Abstract] OR "Nivolumab"[Title/Abstract] OR "Tislelizumab"[Title/Abstract] OR "Ipilimumab"[Title/Abstract] OR "Envafolimab"[Title/Abstract] | 208,202 |
| 5 | "Immunotherapy"[MeSH Terms] | 348,188 |
| 4 | "Rectal Neoplasms"[MeSH Terms] OR "Rectal Neoplasms"[Title/Abstract] OR "neoplasm rectal"[Title/Abstract] OR "rectal neoplasm"[Title/Abstract] OR "neoplasms rectal"[Title/Abstract] OR "rectum neoplasms"[Title/Abstract] OR "neoplasm rectum"[Title/Abstract] OR "rectum neoplasm"[Title/Abstract] OR "rectal tumors"[Title/Abstract] OR "rectal tumor"[Title/Abstract] OR "tumor rectal"[Title/Abstract] OR "cancer of rectum"[Title/Abstract] OR "rectum cancers"[Title/Abstract] OR "cancer of the rectum"[Title/Abstract] OR "rectal cancer"[Title/Abstract] OR "cancer rectal"[Title/Abstract] OR "rectal cancers"[Title/Abstract] OR "rectum cancer"[Title/Abstract] OR "cancer rectum"[Title/Abstract] OR "colorectal cancer"[Title/Abstract] OR "colorectal neoplasms"[Title/Abstract] | 199,057 |
| 3 | "rectal neoplasms"[Title/Abstract] OR "neoplasm rectal"[Title/Abstract] OR "rectal neoplasm"[Title/Abstract] OR "neoplasms rectal"[Title/Abstract] OR "rectum neoplasms"[Title/Abstract] OR "neoplasm rectum"[Title/Abstract] OR "rectum neoplasm"[Title/Abstract] OR "rectal tumors"[Title/Abstract] OR "rectal tumor"[Title/Abstract] OR "tumor rectal"[Title/Abstract] OR "cancer of rectum"[Title/Abstract] OR "rectum cancers"[Title/Abstract] OR "cancer of the rectum"[Title/Abstract] OR "rectal cancer"[Title/Abstract] OR "cancer rectal"[Title/Abstract] OR "rectal cancers"[Title/Abstract] OR "rectum cancer"[Title/Abstract] OR "cancer rectum"[Title/Abstract] OR "colorectal cancer"[Title/Abstract] OR "colorectal neoplasms"[Title/Abstract] | 173,933 |
| 1 | "Rectal Neoplasms"[MeSH Terms] | 56,128 |

**Web of Science**

| No. | Search Details | Results |
| --- | --- | --- |
| 1 | (((((((((((((((((((TS=("Rectal Tumor")) OR TS=("Neoplasm, Rectal" )) OR TS=("Rectal Neoplasm")) OR TS=("Neoplasms, Rectal")) OR TS=("Rectum Neoplasms")) OR TS=("Neoplasm, Rectum")) OR TS=("Rectum Neoplasm")) OR TS=("Rectal Tumors")) OR TS=("Rectal Neoplasms")) OR TS=("Tumor, Rectal")) OR TS=("Cancer of Rectum")) OR TS=("Rectum Cancers")) OR TS=("Cancer of the Rectum")) OR TS=("Rectal Cancer")) OR TS=("Cancer, Rectal")) OR TS=("Rectal Cancers")) OR TS=("Rectum Cancer")) OR TS=("Cancer, Rectum")) OR TS=("Colorectal cancer")) OR TS=("colorectal neoplasms") | 430579 |
| 2 | (((((((((((((((((((((((((((((((((((((((TS=(immunotherapy)) OR TS=(immunotherapies)) OR TS=("immunotherapeutic agents")) OR TS=(immunotherapeutic)) OR TS=("checkpoint inhibitor")) OR TS=("checkpoint inhibitors")) OR TS=("immune checkpoint inhibitors")) OR TS=("checkpoint blockade")) OR TS=("checkpoint blockades")) OR TS=("programmed cell death protein 1")) OR TS=("programmed cell death 1")) OR TS=("programmed death 1")) OR TS=("programmed death protein 1")) OR TS=("programmed cell death receptor-1 inhibitor")) OR TS=("programmed cell death receptor-1 inhibitors")) OR TS=("programmed cell death 1 receptor inhibitor")) OR TS=("programmed cell death 1 receptor inhibitors")) OR TS=("anti-pd-1 antibody")) OR TS=("anti-pd-1 antibodies")) OR TS=("programmed cell death 1 ligand 1")) OR TS=("programmed death 1 ligand 1")) OR TS=("programmed death ligand 1")) OR TS=("PD-1")) OR TS=( PD1 )) OR TS=("PD-L1")) OR TS=( PDL1)) OR TS=("anti-pd-l1 antibody")) OR TS=("anti-pd-l1 antibodies")) OR TS=("anti-pd-l1 inhibitor")) OR TS=("anti-pd-l1 inhibitors")) OR TS=("PD-1 inhibitors")) OR TS=("PD-L1 inhibitors")) OR TS=("CTLA-4 inhibitors")) OR TS=("LAG-3 inhibitors")) OR TS=("TIM-3 inhibitors")) OR TS=(Pembrolizumab )) OR TS=(Nivolumab)) OR TS=(Tislelizumab)) OR TS=(Ipilimumab)) OR TS=(Envafolimab) | 446859 |
| 3 | ((((((((((((((((((((TS=("neoadjuvant therapy ")) OR TS=(neoadjuvant )) OR TS=(preoperative)) OR TS=(presurgical )) OR TS=(presurgery )) OR TS=(preresection )) OR TS=(preresectional)) OR TS=(preoperation )) OR TS=(preoperations )) OR TS=("pre-operative")) OR TS=("pre-surgical ")) OR TS=("pre-surgery")) OR TS=("pre-operation")) OR TS=("pre-operation")) OR TS=("pre-resection")) OR TS=("before surgery")) OR TS=("before resection")) OR TS=("before operation")) OR TS=("prior to resection")) OR TS=("prior to surgery")) OR TS=("prior to operation") | 652048 |
| 4 | #3 AND #2 | 9960 |
| 5 | #4 AND #1 | 913 |

**Cochrane**

| No. | Search Details | Results |
| --- | --- | --- |
| #1 | MeSH descriptor: [Rectal Neoplasms] explode all trees | 2883 |
| #2 | (Neoplasm, Rectal):ab,ti,kw OR (Rectal Neoplasm):ab,ti,kw OR (Neoplasms, Rectal):ab,ti,kw OR (Rectum Neoplasm):ab,ti,kw OR (Neoplasm, Rectum):ab,ti,kw OR (Rectal Tumor):ab,ti,kw OR (Rectal Tumors):ab,ti,kw OR (Rectal Neoplasms):ab,ti,kw OR (Tumor, Rectal):ab,ti,kw OR (Cancer of Rectum):ab,ti,kw OR (Rectum Cancers):ab,ti,kw OR (Cancer of the Rectum):ab,ti,kw OR (Rectal Cancer):ab,ti,kw OR (Cancer, Rectal):ab,ti,kw OR (Rectal Cancers):ab,ti,kw OR (Rectum Cancer):ab,ti,kw OR (Cancer, Rectum):ab,ti,kw OR (Colorectal cancer):ab,ti,kw OR (colorectal neoplasms):ab,ti,kw | 26465 |
| #3 | #1 OR #2 | 26628 |
| #4 | MeSH descriptor: [Immunotherapy] explode all trees | 12175 |
| #5 | (immunotherapies):ab,ti,kw OR (immunotherapeutic):ab,ti,kw OR (immunotherapeutic agents):ab,ti,kw OR (checkpoint inhibitor):ab,ti,kw OR (checkpoint inhibitors ):ab,ti,kw OR (immune checkpoint inhibitors):ab,ti,kw OR (checkpoint blockade):ab,ti,kw OR (checkpoint blockades):ab,ti,kw OR (programmed cell death protein 1):ab,ti,kw OR (programmed cell death 1):ab,ti,kw OR (programmed death 1):ab,ti,kw OR (programmed death protein 1):ab,ti,kw OR (programmed cell death receptor-1 inhibitor):ab,ti,kw OR (programmed cell death receptor-1 inhibitors):ab,ti,kw OR (programmed cell death 1 receptor inhibitor):ab,ti,kw OR (programmed cell death 1 receptor inhibitors):ab,ti,kw OR (anti-pd-1 antibody):ab,ti,kw OR (anti-pd-1 antibodies):ab,ti,kw OR (programmed cell death 1 ligand 1):ab,ti,kw OR (programmed death 1 ligand 1):ab,ti,kw OR (programmed death ligand 1):ab,ti,kw OR (PD-1):ab,ti,kw OR (PD1):ab,ti,kw OR (PD-L1):ab,ti,kw OR (PDL1):ab,ti,kw OR (anti-pd-l1 antibody):ab,ti,kw OR (anti-pd-l1 antibodies ):ab,ti,kw OR (anti-pd-l1 inhibitor):ab,ti,kw OR (anti-pd-l1 inhibitors):ab,ti,kw OR (PD-1 inhibitors):ab,ti,kw OR (PD-L1 inhibitors):ab,ti,kw OR (CTLA-4 inhibitors):ab,ti,kw OR (LAG-3 inhibitors):ab,ti,kw OR (TIM-3 inhibitors):ab,ti,kw OR (Pembrolizumab):ab,ti,kw OR (Nivolumab):ab,ti,kw OR (Tislelizumab):ab,ti,kw OR (Ipilimumab):ab,ti,kw OR (Envafolimab):ab,ti,kw | 12588 |
| #6 | #4 OR #5 | 24329 |
| #7 | MeSH descriptor: [Neoadjuvant Therapy] explode all trees | 2641 |
| #8 | (neoadjuvant):ab,ti,kw OR (preoperative):ab,ti,kw OR (presurgical):ab,ti,kw OR (presurgery):ab,ti,kw OR (preresection):ab,ti,kw OR (preresectional):ab,ti,kw OR (preoperation):ab,ti,kw OR (preoperations):ab,ti,kw OR (pre-operative):ab,ti,kw OR (pre-surgical):ab,ti,kw OR (pre-surgery):ab,ti,kw OR (pre-operation):ab,ti,kw OR (pre-operations):ab,ti,kw OR (pre-resection):ab,ti,kw OR (before surgery):ab,ti,kw OR (before resection):ab,ti,kw OR (before operation):ab,ti,kw OR (prior to resection):ab,ti,kw OR (prior to surgery):ab,ti,kw OR (prior to operation):ab,ti,kw | 127745 |
| #9 | #7 OR #8 | 127745 |
| #10 | #3 AND #6 AND #9 | 128 |

**EMBASE**

| No. | Search Details | Results |
| --- | --- | --- |
| #11 | #9 AND #10 | 876 |
| #10 | #3 AND #6 | 10189 |
| #9 | #7 OR #8 | 708059 |
| #8 | neoadjuvant':ab,ti OR 'preoperative':ab,ti OR 'pre-operative':ab,ti OR 'preoperation':ab,ti OR 'preoperations':ab,ti OR 'pre-operation':ab,ti OR 'pre-operations':ab,ti OR 'presurgical':ab,ti OR 'pre-surgical':ab,ti OR 'presurgery':ab,ti OR 'pre-surgery':ab,ti OR 'preresection':ab,ti OR 'pre-resection':ab,ti OR 'preresectional':ab,ti OR 'before surgery':ab,ti OR 'before resection':ab,ti OR 'before operation':ab,ti OR 'prior to resection':ab,ti OR 'prior to surgery':ab,ti OR 'prior to operation':ab,ti | 695471 |
| #7 | 'neoadjuvant therapy'/exp | 59859 |
| #6 | #4 OR #5 | 458264 |
| #5 | immunotherapies':ab,ti OR 'immunotherapeutic':ab,ti OR 'immunotherapeutic agents':ab,ti OR 'checkpoint inhibitor':ab,ti OR 'checkpoint inhibitors':ab,ti OR 'immune checkpoint inhibitors':ab,ti OR 'checkpoint blockade':ab,ti OR 'checkpoint blockades':ab,ti OR 'programmed cell death protein 1':ab,ti OR 'programmed cell death 1':ab,ti OR 'programmed death 1':ab,ti OR 'programmed death protein 1':ab,ti OR 'programmed cell death receptor-1 inhibitor':ab,ti OR 'programmed cell death receptor-1 inhibitors':ab,ti OR 'programmed cell death 1 receptor inhibitor':ab,ti OR 'programmed cell death 1 receptor inhibitors':ab,ti OR 'anti-pd-1 antibody':ab,ti OR 'anti-pd-1 antibodies':ab,ti OR 'programmed cell death 1 ligand 1':ab,ti OR 'programmed death 1 ligand 1':ab,ti OR 'programmed death ligand 1':ab,ti OR 'pd-1':ab,ti OR 'pd1':ab,ti OR 'pd-l1':ab,ti OR 'pdl':ab,ti OR 'anti-pd-l1 antibody':ab,ti OR 'anti-pd-l1 antibodies':ab,ti OR 'anti-pd-l1 inhibitor':ab,ti OR 'anti-pd-l1 inhibitors':ab,ti OR 'pd-1 inhibitors':ab,ti OR 'pd-l1 inhibitors':ab,ti OR 'ctla-4 inhibitors':ab,ti OR 'lag-3 inhibitors':ab,ti OR 'tim-3 inhibitors':ab,ti OR 'pembrolizumab':ab,ti OR 'nivolumab':ab,ti OR 'tislelizumab':ab,ti OR 'ipilimumab':ab,ti OR 'envafolimab':ab,ti | 193660 |
| #4 | 'immunotherapy'/exp | 342974 |
| #3 | #1 OR #2 | 285554 |
| #2 | 'neoplasm, rectal':ab,ti OR 'rectal neoplasm':ab,ti OR 'neoplasms, rectal':ab,ti OR 'rectum neoplasms':ab,ti OR 'neoplasm, rectum':ab,ti OR 'rectum neoplasm':ab,ti OR 'rectal tumors':ab,ti OR 'rectal neoplasms':ab,ti OR 'tumor, rectal':ab,ti OR 'cancer of rectum':ab,ti OR 'rectum cancers':ab,ti OR 'cancer of the rectum':ab,ti OR 'rectal cancer':ab,ti OR 'cancer, rectal':ab,ti OR 'rectal cancers':ab,ti OR 'rectum cancer':ab,ti OR 'cancer, rectum':ab,ti OR 'colorectal cancer':ab,ti OR 'colorectal neoplasms':ab,ti | 250172 |
| #1 | 'rectum tumor'/exp | 87336 |
