# Supplementary figures and images for: Neoadjuvant immunotherapy for DNA mismatch repair proficient/microsatellite stable non-metastatic rectal cancer: a systematic review and meta-analysis
Source: Front Immunol. 2025 Jan 27;16:1523455. doi: 10.3389/fimmu.2025.1523455 (PMC11808008; doi:10.3389/fimmu.2025.1523455)

**Trim and Fill Method**

**irAEs rates**


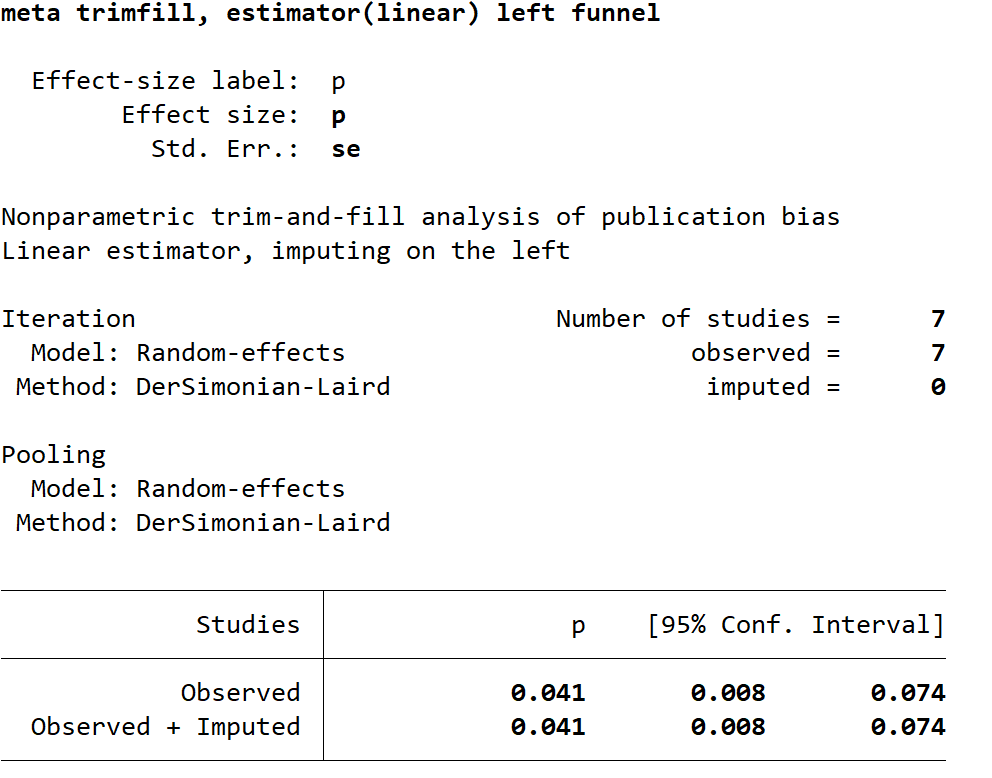

Supplement: Supplementary file 3 [file DataSheet3.docx]
