# Supplementary material for: Neoadjuvant immunotherapy for DNA mismatch repair proficient/microsatellite stable non-metastatic rectal cancer: a systematic review and meta-analysis
Source: Front Immunol. 2025 Jan 27;16:1523455. doi: 10.3389/fimmu.2025.1523455 (PMC11808008; doi:10.3389/fimmu.2025.1523455)
Supplement: Supplementary file 4 [file DataSheet4.docx]

**Supplementary Figure**

**
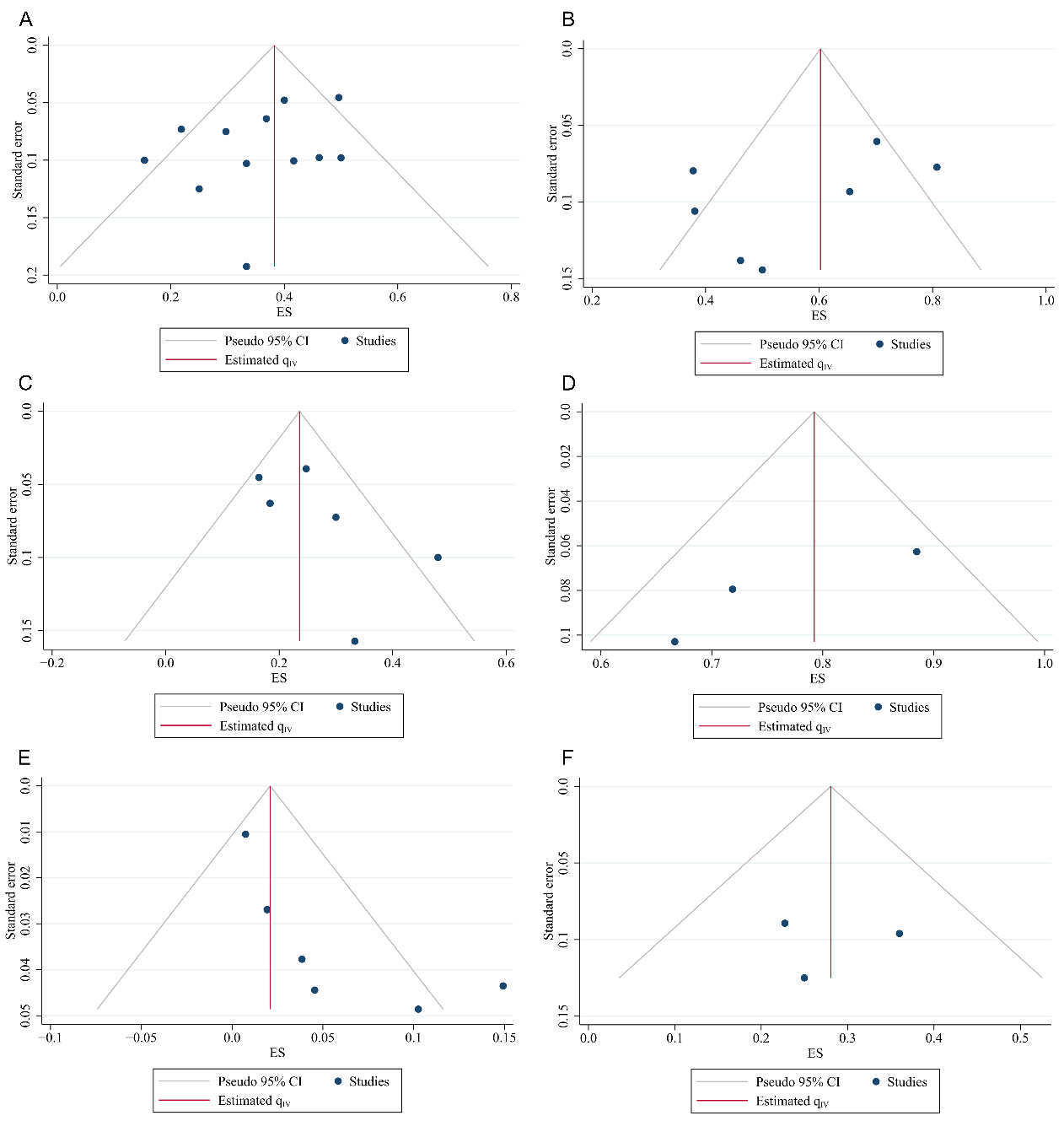
**

Supplementary Figure 1 Funnel plots of (A) pCR, (B) MPR, (C) cCR, (D) anus preservation rates, (E) incidence of irAEs≥3 grades and (F) incidence of TRAEs ≥3 grades.


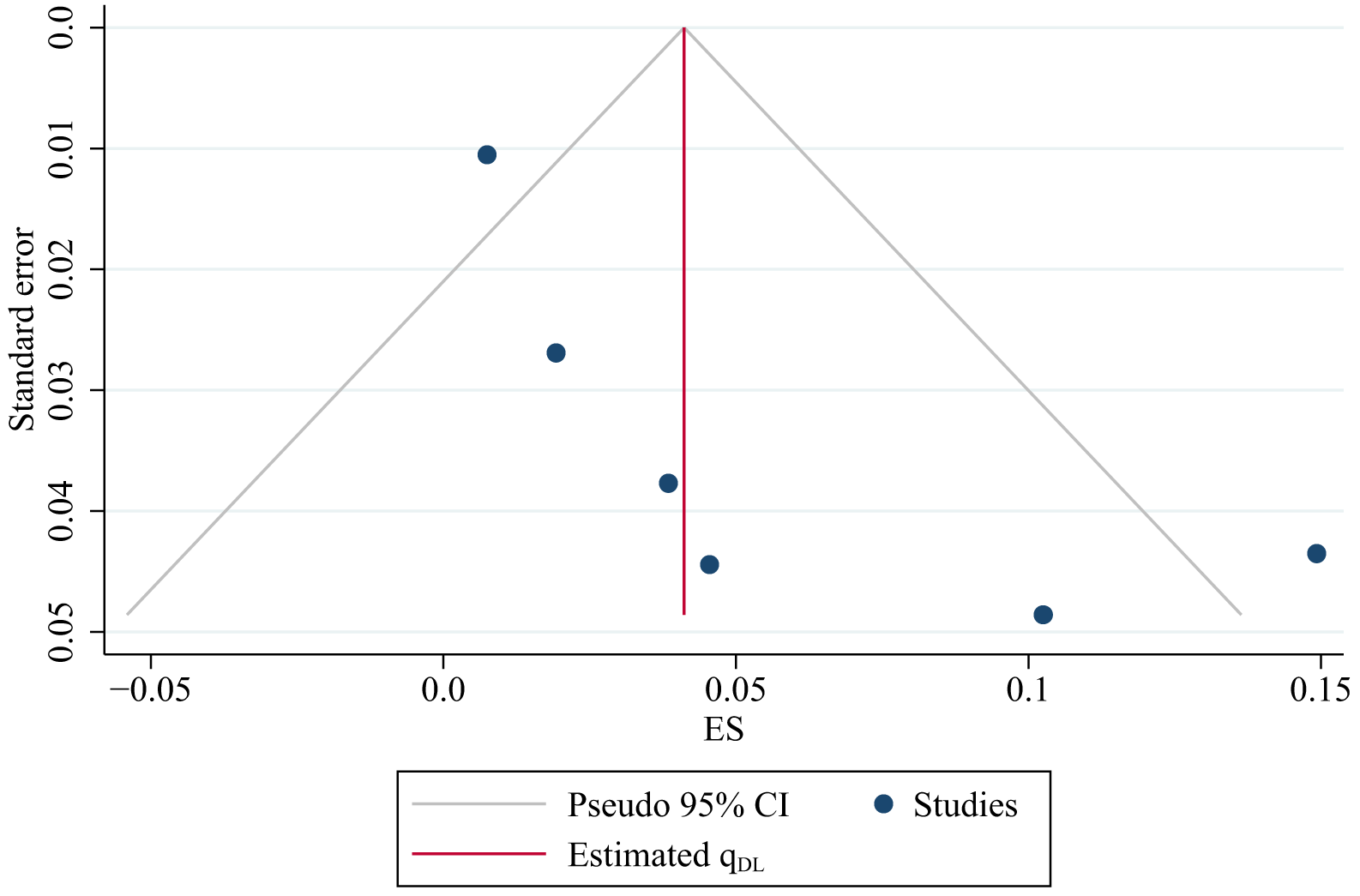


Supplementary Figure 2 Funnel plots of irAEs from trim and fill method.


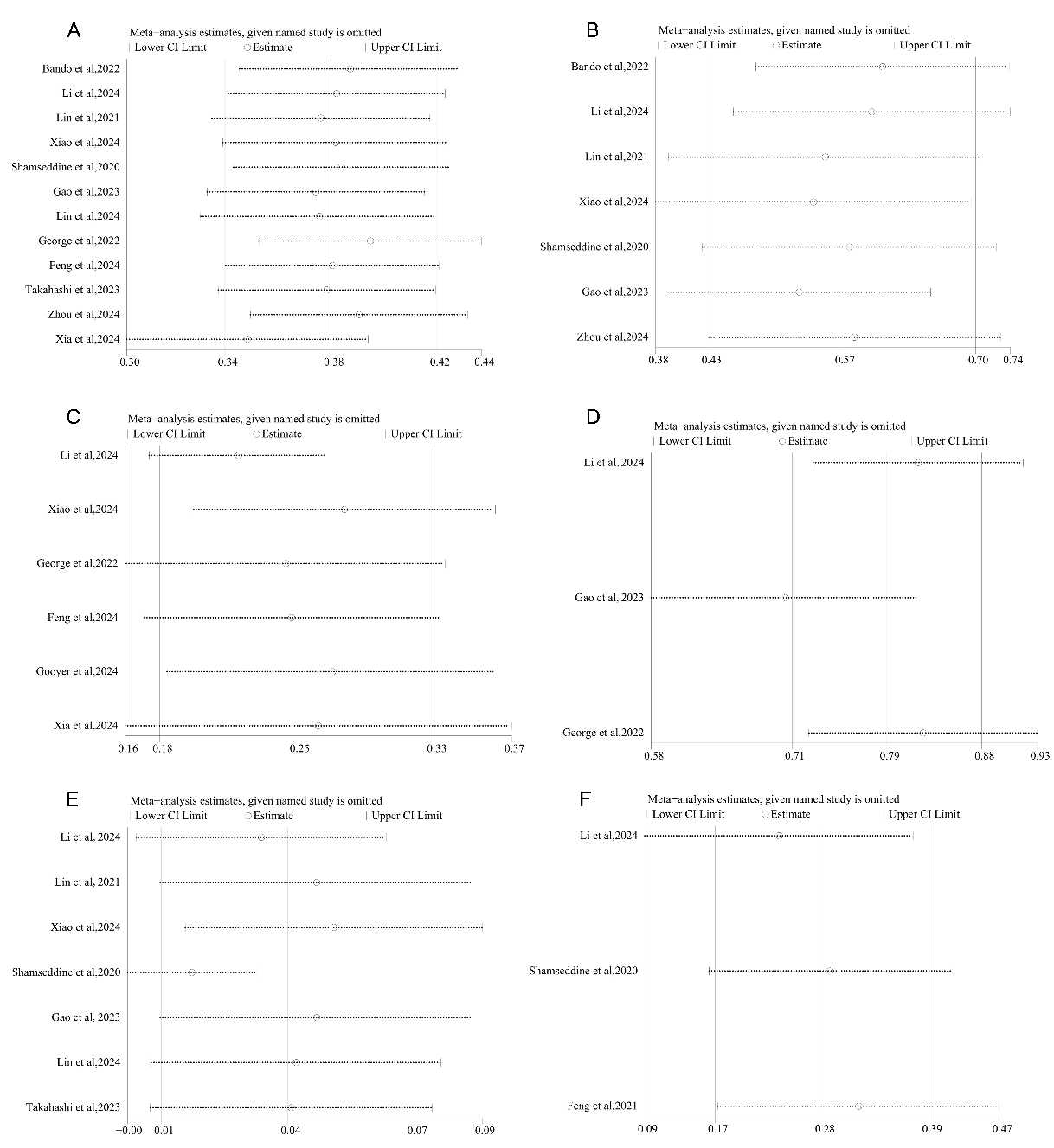


Supplementary Figure 3 Sensitivity analysis. Sensitivity analysis of pCR (A), MPR (B), cCR (C), anus preservation rate (D), irAEs (E) and TRAEs (F).


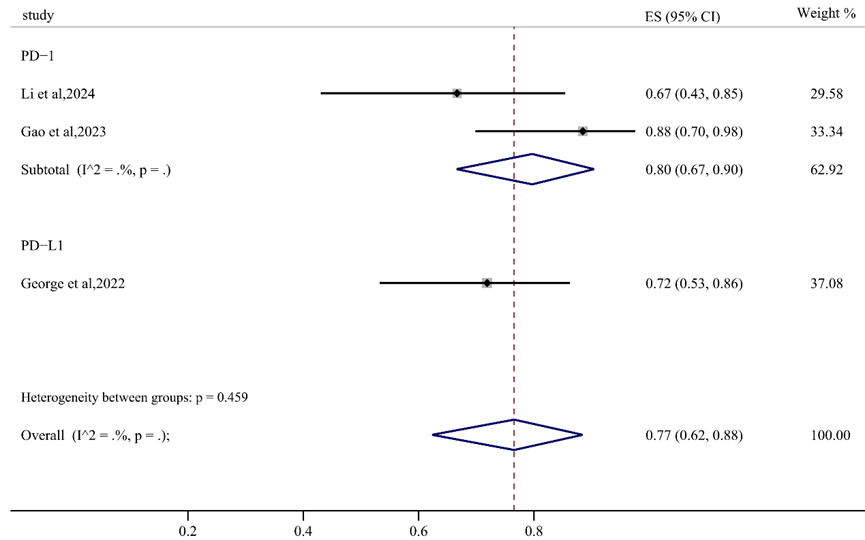


Supplementary Figure 4 The forest figure of anus preservation rate based on PD-1/PD-L1 checkpoint inhibitor subgroup analysis.


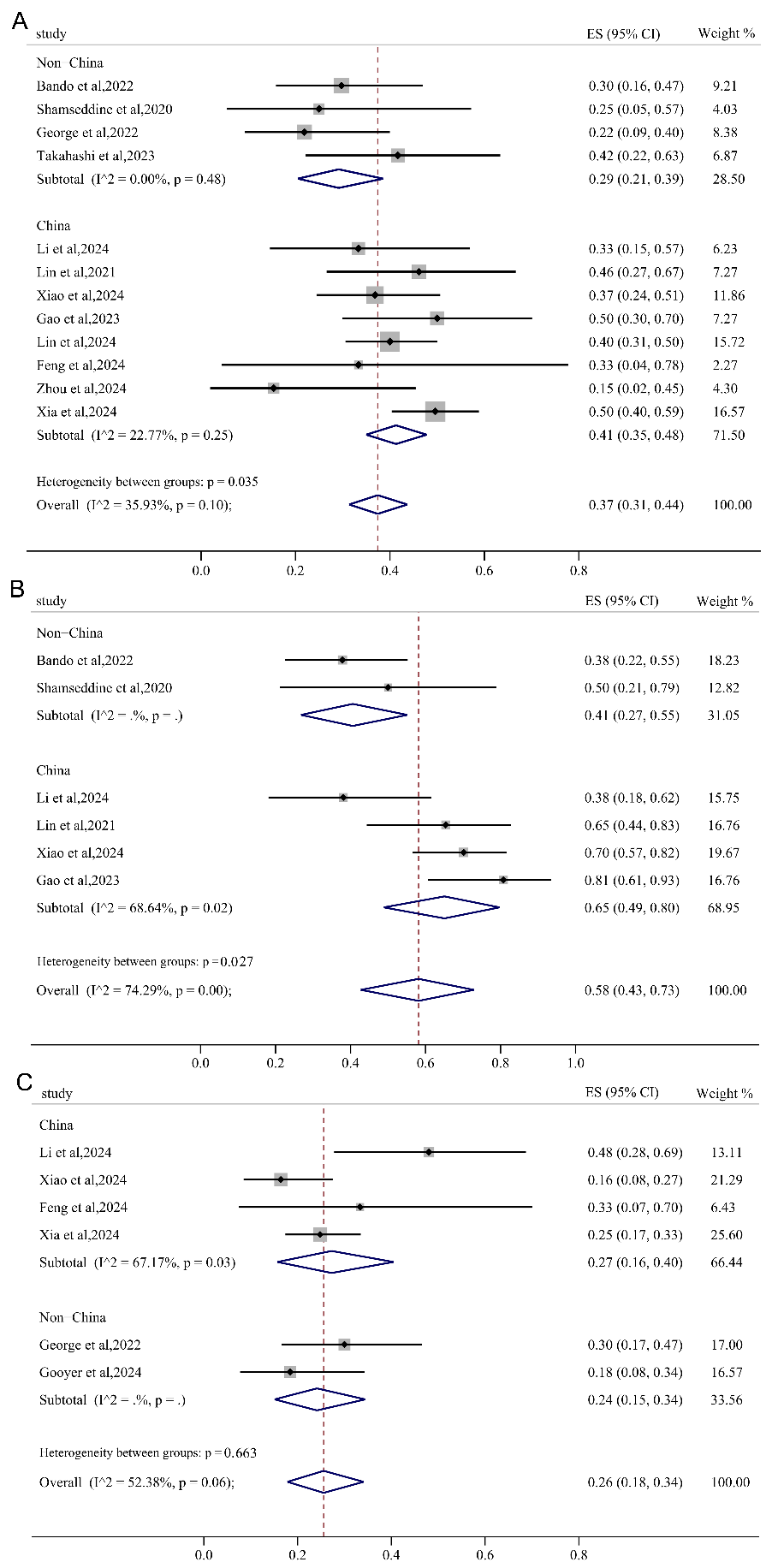


Supplementary Figure 5 The forest figure of response rate (pCR, MPR and cCR) based on region subgroup analysis. (A) pCR rate based on region subgroup analysis; (B) MPR rate based on region subgroup analysis; (C) cCR based on region subgroup analysis.
