# Supplementary material for: Neoadjuvant immunotherapy for DNA mismatch repair proficient/microsatellite stable non-metastatic rectal cancer: a systematic review and meta-analysis
Source: Front Immunol. 2025 Jan 27;16:1523455. doi: 10.3389/fimmu.2025.1523455 (PMC11808008; doi:10.3389/fimmu.2025.1523455)
Supplement: Supplementary file 6 [file Table2.docx]

**Supplementary table 2 Immunotherapy-related adverse effects**

| Study | Pruritus | | Dermatitis | | Endocrine disorders | | Gastrointestinal disorders | | Reactive cutaneous capillary endothelial proliferation | | Sum | I-II | III-IV |
| --- | --- | --- | --- | --- | --- | --- | --- | --- | --- | --- | --- | --- | --- |
|  | I-II | III-IV | I-II | III-IV | I-II | III-IV | I-II | III-IV | I-II | III-IV |  |  |  |
| Bando et al, 2022 | 8 | 0 | NA | NA | 5 | 0 | 8 | 2 | NA | NA | 21 | 17 | 4 |
| Li et al, 2024 | NA | 0 | NA | 0 | NA | 0 | NA | 0 | 18 | 0 | NA | NA | 0 |
| Lin et al, 2021 | NA | 0 | NA | 0 | NA | 0 | NA | 0 | NA | 0 | NA | NA | 0 |
| Xiao et al, 2024 | NA | NA | NA | NA | NA | NA | NA | NA | NA | NA | NA | NA | 10 |
| Shamseddine et al, 2020 | 0 | 0 | 0 | 0 | 0 | 0 | 0 | 0 | 0 | 0 | 0 | 0 | 0 |
| Gao et al, 2023 | 0 | 0 | 0 | 0 | 2 | 0 | 0 | 0 | 0 | 0 | 5 | 4 | 1 |
| Lin et al, 2024 | NA | 0 | NA | 0 | NA | 0 | NA | 0 | NA | 0 | NA | NA | NA |
| George et al, 2022 | NA | NA | NA | NA | NA | NA | NA | NA | NA | NA | NA | NA | NA |
| Feng et al, 2024 | NA | 0 | 0 | 1 | NA | 0 | NA | 0 | NA | 0 | 3 | 2 | 1 |
| Takahashi et al, 2023 | NA | NA | NA | NA | NA | NA | NA | NA | NA | NA | NA | NA | NA |
| Gooyer et al, 2024 | NA | NA | NA | NA | NA | NA | NA | NA | NA | NA | NA | NA | 3 |
| Zhou et al, 2024 | NA | NA | NA | NA | NA | NA | NA | NA | NA | NA | NA | NA | NA |
| Xia et al, 2024 | NA | NA | NA | NA | NA | NA | NA | NA | NA | NA | NA | NA | NA |
