# Supplementary material for: Neoadjuvant immunotherapy for DNA mismatch repair proficient/microsatellite stable non-metastatic rectal cancer: a systematic review and meta-analysis
Source: Front Immunol. 2025 Jan 27;16:1523455. doi: 10.3389/fimmu.2025.1523455 (PMC11808008; doi:10.3389/fimmu.2025.1523455)
Supplement: Supplementary file 7 [file Table3.docx]

**Supplementary table 3 Clinical stage**

| Study | clinical T stage | | | | | | clinical N stage | | clinical M stage | | Clinical stage | | | |
| --- | --- | --- | --- | --- | --- | --- | --- | --- | --- | --- | --- | --- | --- | --- |
|  | Tis | T0 | T1 | T2 | T3 | T4 | N0 | N+ | M0 | M1 | I | II | III | IV |
| Bando et al, 2022 | 0 | 0 | 0 | 0 | 34 | 5 | 30 | 9 | 39 | 0 | 0 | 30 | 9 | 0 |
| Li et al, 2024 | 0 | 0 | 0 | 0 | 20 | 5 | NA | 25 | 025 | 0 | 0 | 0 | 25 | 0 |
| Lin et al, 2021 | 0 | 0 | 0 | 0 | NA | NA | NA | NA | 26 | 0 | 0 | NA | NA | 0 |
| Xiao et al, 2024 |  |  | 0 | 2 | 30 | 35 | 10 | 57 | 134 | 0 | 0 | 10 | 57 | 0 |
| Shamseddine et al, 2020 | 0 | 0 | 0 | 10 | 8 | 0 | 13 | 5 | 13 | 0 | 8 | 5 | 5 | 0 |
| Gao et al, 2023 | 0 | 0 | 0 | 4 | 19 | 3 | 12 | 14 | 16 | 0 | 0 | NA | NA | 0 |
| Lin et al, 2024 | 0 | 0 | 0 | 7 | 72 | 34 | 14 | 99 | 0 | 0 | 0 | 17 | 96 | 0 |
| George et al, 2022 | 0 | 0 | 0 | NA | NA | NA | NA | NA | 45 | 0 | 0 | NA | NA | 0 |
| Feng et al, 2024 | 0 | 0 | NA | NA | NA | NA | NA | NA | 22 | 0 | 0 | 2 | 20 | 0 |
| Takahashi et al, 2023 | 0 | 0 | 0 | 0 | NA | NA | NA | NA | 24 | 0 | 0 | NA | NA | 0 |
| Gooyer et al, 2024 | 0 | 0 | NA | NA | 27 | 1 | 23 | 21 | 38 | 0 | 0 | NA | NA | 0 |
| Zhou et al, 2024 | 0 | 0 | 0 | 0 | 11 | 5 | 1 | 15 | 16 | 0 | 0 | NA | NA | 0 |
| Xia et al, 2024 | 0 | 0 | 0 | 0 | NA | NA | NA | NA | 121 | 0 | 0 | NA | NA | 0 |
