# Supplementary material for: Neoadjuvant immunotherapy for DNA mismatch repair proficient/microsatellite stable non-metastatic rectal cancer: a systematic review and meta-analysis
Source: Front Immunol. 2025 Jan 27;16:1523455. doi: 10.3389/fimmu.2025.1523455 (PMC11808008; doi:10.3389/fimmu.2025.1523455)
Supplement: Supplementary file 8 [file Table4.docx]

**Supplementary table 4 MINORS score**

| Study | A | B | C | D | E | F | G | H | Score |
| --- | --- | --- | --- | --- | --- | --- | --- | --- | --- |
| Bando et al, 2022 | 2 | 2 | 2 | 2 | 2 | 2 | 2 | 0 | 14 |
| Li et al, 2024 | 2 | 2 | 2 | 2 | 2 | 2 | 2 | 0 | 14 |
| Lin et al, 2021 | 2 | 2 | 2 | 2 | 2 | 2 | 2 | 2 | 16 |
| Xiao et al, 2024 | 2 | 2 | 2 | 2 | 2 | 2 | 2 | 2 | 16 |
| Shamseddine et al, 2020 | 2 | 2 | 2 | 2 | 2 | 2 | 2 | 0 | 14 |
| Gao et al, 2023 | 2 | 2 | 2 | 2 | 2 | 2 | 2 | 0 | 14 |
| Lin et al, 2024 | 2 | 2 | 2 | 2 | 2 | 2 | 2 | 2 | 16 |
| George et al, 2022 | 2 | 2 | 2 | 2 | 2 | 2 | 2 | 0 | 14 |
| Feng et al, 2024 | 2 | 2 | 2 | 1 | 2 | 2 | 2 | 0 | 13 |
| Takahashi et al, 2023 | 2 | 2 | 2 | 1 | 2 | 2 | 2 | 0 | 13 |
| Gooyer et al, 2024 | 2 | 2 | 2 | 2 | 2 | 2 | 2 | 0 | 14 |
| Zhou et al, 2024 | 2 | 2 | 2 | 1 | 2 | 2 | 2 | 0 | 13 |
| Xia et al, 2024 | 2 | 2 | 2 | 2 | 2 | 2 | 2 | 2 | 16 |

Note:

A: stated aim of the study; B: Inclusion of consecutive patients; C: Prospective collection of data; D: Endpoint appropriate to the study aim; E: Unbiased evaluation of endpoints; F: Follow-up period appropriate to the major endpoint; G: Loss to follow up not exceeding 5%; H: Prospective calculation of the sample size.
